# Immunosuppressive PLGA TGF-β1 Microparticles Induce Polyclonal and Antigen-Specific Regulatory T Cells for Local Immunomodulation of Allogeneic Islet Transplants

**DOI:** 10.3389/fimmu.2021.653088

**Published:** 2021-05-27

**Authors:** Ying Li, Anthony W. Frei, Irayme M. Labrada, Yanan Rong, Jia-Pu Liang, Magdalena M. Samojlik, Chuqiao Sun, Steven Barash, Benjamin G. Keselowsky, Allison L. Bayer, Cherie L. Stabler

**Affiliations:** ^1^ J. Crayton Pruitt Family Department of Biomedical Engineering, University of Florida, Gainesville, FL, United States; ^2^ Graduate Program in Biomedical Sciences, College of Medicine, University of Florida, Gainesville, FL, United States; ^3^ University of Florida Diabetes Institute, Gainesville, FL, United States; ^4^ Diabetes Research Institute, University of Miami, Miami, FL, United States; ^5^ Department of Microbiology and Immunology, University of Miami, Miami, FL, United States

**Keywords:** biomaterials, immunomodulatory, extrahepatic islet transplantation, type 1 diabetes, local drug delivery

## Abstract

Allogeneic islet transplantation is a promising cell-based therapy for Type 1 Diabetes (T1D). The long-term efficacy of this approach, however, is impaired by allorejection. Current clinical practice relies on long-term systemic immunosuppression, leading to severe adverse events. To avoid these detrimental effects, poly(lactic-co-glycolic acid) (PLGA) microparticles (MPs) were engineered for the localized and controlled release of immunomodulatory TGF-β1. The *in vitro* co-incubation of TGF-β1 releasing PLGA MPs with naïve CD4^+^ T cells resulted in the efficient generation of both polyclonal and antigen-specific induced regulatory T cells (iTregs) with robust immunosuppressive function. The co-transplantation of TGF-β1 releasing PLGA MPs and Balb/c mouse islets within the extrahepatic epididymal fat pad (EFP) of diabetic C57BL/6J mice resulted in the prompt engraftment of the allogenic implants, supporting the compatibility of PLGA MPs and local TGF-β1 release. The presence of the TGF-β1-PLGA MPs, however, did not confer significant graft protection when compared to untreated controls, despite measurement of preserved insulin expression, reduced intra-islet CD3^+^ cells invasion, and elevated CD3^+^Foxp3^+^ T cells at the peri-transplantation site in long-term functioning grafts. Examination of the broader impacts of TGF-β1/PLGA MPs on the host immune system implicated a localized nature of the immunomodulation with no observed systemic impacts. In summary, this approach establishes the feasibility of a local and modular microparticle delivery system for the immunomodulation of an extrahepatic implant site. This approach can be easily adapted to deliver larger doses or other agents, as well as multi-drug approaches, within the local graft microenvironment to prevent transplant rejection.

## Introduction

Type 1 Diabetes (T1D) is an autoimmune disease caused by the selective destruction of insulin-producing pancreatic beta cells, resulting in persistent hyperglycemia (1). Exogenous insulin delivery is currently the primary clinical treatment for T1D; however, it is not a cure, as less than half of adults with T1D achieve recommended glycemic control targets (2). Alternatively, clinical islet transplantation (CIT) via intraportal infusion is a potentially curative therapy, as engrafted, viable islets can provide durable and physiological glycemic control (3). While promising, the widespread application of CIT is limited by several factors, including donor cell shortage, adverse effects of systemic immunosuppression, and host-mediated immune rejection (4).

Allogeneic rejection refers to the recognition and clearance of cells or tissues sourced from a genetically different donor(s) of the same species (5). Following allo-islet transplantation, post-surgical inflammatory signals recruit host antigen-presenting cells (APCs) followed by adaptive immune cells, which recognize allogeneic antigens of the transplanted islets and initiate adaptive effector pathways (5–7). Recipient CD4^+^ T cells are key players in these processes, as they facilitate antigen-specific immune responses, *e.g.*, activating donor-specific cytolytic CD8^+^ T cells and B cell-mediated alloantibodies production, that aggressively destroy the islet graft (5–7).

Due to the multiple avenues in which alloreactive immune responses occur, current clinical practice in allograft transplantation relies heavily on long-term systemic immunosuppression (7–9). Typical CIT immunosuppressive regimens consist of an induction phase of T cell depletion with antithymocyte globulin, followed by a maintenance phase to suppress T cell activation and proliferation *via* tacrolimus, rapamycin, and daclizumab therapies (10, 11). Despite improvements in immunosuppressive regimens, most CIT recipients do not achieve long-term insulin independence due to smoldering host-versus-graft immune responses (12). Furthermore, long-term systemic immunosuppression elevates the recipients’ risk of opportunistic infections and cancer, imparts negative impacts on islet graft function, and limits broader implementation of CIT.

An alternative approach to attenuate allograft rejection is through the establishment of a local immunosuppressive or immunotolerant environment that selectively favors the engraftment of the foreign-sourced islets (13). In practice, the acceptance of a foreign-sourced graft can be promoted *via* the local delivery of immunomodulatory and suppressive factors (e.g., TGF-β1, IL-2, IL-10, IDO-1, etc.) (14–16), which provide instructive cues to key immune players, e.g. promoting CD4^+^ T cells differentiation towards a regulatory phenotype and/or inhibiting dendritic cell maturation (17–20).

The immunomodulatory cytokine TGF-β1 is known for its pleiotropic functions in regulating a broad range of immune processes, particularly in promoting peripheral T cell tolerance and suppressing effector functions (21, 22). For example, TGF-β1 signaling is an essential component for the development of immunotolerance, where it supports both central and peripheral regulatory cell phenotypes, e.g., natural T regulatory cells (nTregs). Post-development, TGF-β1 regulates peripheral T cell tolerance *via* multiple fronts. For example, TGF-β1 is a potent inhibitor of CD8^+^ T cell activation and effector function, resulting in substantial decreases in granzyme B, IL-2, IFN-γ, and other cytolytic molecules (21, 23). In addition, TGF-β1 is hypothesized to support the differentiation of naïve CD4^+^ T cells into induced regulatory T cells (iTregs) within peripheral tissues (21, 24). Due to its potency in the induction of regulatory immune responses, TGF-β1 has been used to generate polyclonal or insulin-specific regulatory T cells for adoptive Treg therapy for T1D (25, 26).

Despite the promise of TGF-β1 in tolerance induction and immunosuppression, the delivery of soluble TGF-β1 is restricted by its short half-life (27), off-target effects (18), and potential to induce fibrosis at high doses (28). Incorporating this agent within a biomaterial microparticle system can support targeted controlled release of TGF-β1, providing ease in delivery and localization within the islet transplant site. Poly(lactic-co-glycolic) acid (PLGA) is a biodegradable polymer that is commonly leveraged as a drug-eluting biomaterial for the controlled and sustained release of agents *via* hydrolysis and bulk erosion (29). The biocompatibility and feasibility of using PLGA as a drug delivery platform for local TGF-β1 delivery have been established (15, 17, 30). For immunomodulation, TGF-β1-releasing PLGA materials have generated regulatory T cells and dendritic cells *in vitro*, as well as contributed to the delay in autoimmune progression of T1D and experimental autoimmune encephalomyelitis (EAE) (20, 31, 32). For the protection of allografts, the efficacy of local TGF-β1 released from solid disks within a porous scaffold has been observed, with a modest but significant delay in the rejection of allogenic islet transplants (17). While promising, a macroscale implant lacks the adaptability and scalability for placement within different implant sites or co-injection with islets.

In this study, a PLGA microparticle platform was designed for the localized and controlled release of TGF-β1 to induce Tregs, both *in vitro* and *in vivo*, with the goal of elevating graft tolerance and improving islet transplantation outcomes. Initial work focused on modulating PLGA MP characteristics to tailor TGF-β1 release profiles. The size, release kinetics, and encapsulation efficiency of PLGA microparticle formulations were characterized. Subsequently, the cytocompatibility of the TGF-β1/PLGA MPs and their capacity to generate functional polyclonal and antigen-specific iTreg cells were examined *in vitro*. Finally, TGF-β1/PLGA MPs were co-transplanted with allogeneic islet grafts in a chemically-induced diabetic murine model to characterize the impacts of local immunomodulation on islet engraftment and protection, as well as host cell immunophenotypes. The capacity of TGF-β1/PLGA MPs to promote immunotolerance, as well as their potential localized/systemic immune impacts on the recipients, were also explored and discussed.

## Methods and Materials

### TGF-β1/PLGA Microparticle Fabrication and Characterization

Microparticles made from PLGA loaded with TGF-β1 were fabricated by a double emulsion method. Variations in particle formulations are listed (**Table S1**). PLGA (50:50, 0.45 dL/g, Lakeshore Biomaterials; 50:50, 0.2 dL/g, Sigma; 75:25, 0.2 dL/g Sigma; or 100:0, 0.2 dL/g, Sigma; 100 mg) was dissolved in dichloromethane (20% w/w). The aqueous solution of human recombinant TGF-β1 (2 µg, Peprotech Inc.) with 0.1% BSA carrier protein was added into the PLGA solution and mixed with a homogenizer (Dremel) at 10,000 rpm. Then 4 mL of 2.5% w/v polyvinyl alcohol (PVA, MP) solution was added into the emulsified solution for the second mix at 10,000 rpm. After two emulsion processes, particles were moved into a collection bath of 100 ml 1% w/v PVA solution and stirred at approximately 100 rpm for 24 hours allowing for methylene chloride evaporation and particle stabilization. For formulation E, the PVA collecting bath was enhanced with 2% w/v NaCl. The resulting particles were collected and washed by serial centrifugation in PBS, flash- frozen in liquid nitrogen, dried *via* lyophilization, and stored at -20°C before use. BSA PLGA MPs were made as the vehicle control.

PLGA MPs were characterized by size distribution, release kinetics, encapsulation efficiency, and surface morphology. The size of TGF-β1/PLGA MPs was determined *via* laser diffraction particle size analysis (Coulter LS13320). Microparticle size distribution was further characterized by calculating the polydispersity index (PDI), following established protocol (33). Specifically, polydispersity index (PDI) was calculated as PDI = (σ/2a)^2^, where σ is the standard deviation of the particle size distribution, and a is the mean particle size. The release kinetics of TGF-β1 from PLGA MPs was evaluated by human TGF-β1 ELISA (R&D Systems, Inc.) following the manufacturer’s instruction. TGF-β1 release samples were prepared by incubating 10 mg of MPs in a 1.7 mL low protein binding tube containing 1 mL of PBS with 2% Tween-20 with consistent rotation followed by eluant harvest at designed timepoints. To determine encapsulation efficiency, 10mg of MPs were dissolved in 1mL of 0.2 M NaOH with 5% SDS to disperse the protein into the aqueous phase. Total encapsulated protein was determined *via* a micro-BCA kit (Thermo Fisher Scientific Inc.). Calibration curves were also run using TGF-β1 with 0.1% BSA carrier protein for both the micro BCA and TGF-β1 ELISA kits. To calculate TGF-β1 release and encapsulation efficiency, values was normalized to the proportion of the TGF-β1-BSA stock to account for BSA protein contributions. To examine the surface morphology of PLGA MPs, scanning electron microscopy (SEM) was performed on the drug-loaded particles at different time points (day 0, 7, 14, and 28) during drug release. Images were acquired using an electron microscope (SU5000, Hitachi High Technologies America, Inc.) at 10.0kV at the ICBR Electron Microscopy Core at the University of Florida, Gainesville, FL.

### *In Vitro* iTreg Generation Using TGF-β1 PLGA Microparticles

Naïve CD4^+^ T cells (CD4^+^CD44^low^CD62L^high^) isolated and purified (Mouse naïve CD4^+^ T Cell Isolation Kit, StemCell, Inc.) from the splenocytes of C57BL/6 or OTII mice were used for polyclonal or monoclonal iTreg conversion assay *in vitro*. Naïve CD4^+^ T cells purification was evaluated by the immune staining with Live/Dead^®^ Fixable Near IR dye (Invitrogen), anti-mCD4-PE, anti-mCD62L-PerCP/Cy5.5, anti-mFoxp3-FITC, and anti-mhelios-AF647 (**Table S2**); followed by the flow cytometry analysis of the frequency of viable naïve CD4^+^ T cells (Live/Dead-CD4+CD62L+) and potential contamination of thymic (Live/Dead-CD4+helios+) and natural (Live/Dead-CD4+Foxp3+) Treg cells.

For polyclonal iTreg generation, naïve CD4+ T cells from C57BL/6 mice were activated by anti-CD3/CD28 Dynabeads^®^ (Thermo Fisher Scientific Inc.) at a 3:1 bead to cell ratio, with a titration of TGF-β1/PLGA MPs (1, 3, 10, 33, 100 µg/per 100k naïve CD4^+^ T cells) as immunomodulation. The naïve CD4^+^ T cells were dyed with CellTrace Violet (Thermo Fisher Scientific, Inc.) to measure proliferation, and co-culture was performed in 96 well U-bottom tissue culture treated plate (Corning Inc.) for three days. A titration of soluble TGF-β1 was used as the treatment control, while the BSA PLGA MPs were used as the vehicle controls.

For antigen-specific iTreg conversion, naïve CD4^+^ T cells were sourced from OTII mice, which are clonally specific to ovalbumin 323-339 (OVA_323-339_) peptide in the context of I-A^b^ presentation. Given the antigen specificity, naïve OTII CD4^+^ T cells labeled with CellTrace Violet dye were stimulated with 0.5 µM of OVA_323-339_ peptide (AnaSpec, Inc.) along with mitomycin c treated syngeneic APCs (generated through complement-based T cell depletion) at a 1:1 ratio, with titrated dosages of particle released/soluble TGF-β1 for three days.

Treg induction by TGF-β1/PLGA MPs was evaluated *via* flow cytometry. After three days of induction, CD4^+^ T cells were sequentially stained with Live/Dead^®^ Fixable Near IR dye (Invitrogen), anti-mCD4-PE, anti-mCD25-PE/Cy7, anti-mFoxp3-FITC, and anti-mhelios-AF647 (**Table S2**) for viability and immune phenotyping. Compensation controls were prepared using UltraComp Beads (Invitrogen). Background signals were identified and excluded by fluorescence-minus-one controls. The iTreg conversion rate was quantified as the percentage of proliferating Foxp3^+^ CD4^+^ T cells (**Figure S3**). Data were acquired using BD™ LSRII or FACSCelesta analyzer. Data analysis was performed using FCS Express 6.05 software (De Novo Software).

### iTreg Suppression Assay

iTregs were generated from naïve CD4^+^ T cells of C57BL/6-FIR (Foxp3 induced mRFP) reporter mice using either PLGA MPs released (300 ug/10^5^ CD4^+^ T cells) or soluble TGF-β1(3 ng/mL) for a three-day induction, with 1 × 10^5^ cells and 7.5 µl anti-CD3/CD28 Dynabeads^®^ per well in 96-well U-bottom plates.

Post-induction, iTreg was identified as the LiveDead^-^CD4^+^Foxp3(mRFP)^+^ population and purified by cell sorting (**Figure S4**). The collected iTregs, noted as the suppressor population, were mixed with the CellTrace Violet labeled LiveDead^-^CD4^+^Foxp3(mRFP)^-^ responder population at different ratios (1:16, 1:8, 1:4, 1:2, and 1:1), with anti-CD3 stimulation (2C11, Thermo Fisher Scientific Inc.) and syngeneic APCs, for another three-day co-culture in a 96-well U-bottom plate, as described previously (34, 35). For direct comparison with PLGA/TGF-β1 iTregs, freshly sorted sTGF-β1 iTregs and natural Tregs (nTregs) sourced from B6-FIR mice, were used as controls.

After the three-day co-culture, samples were stained with Live/Dead^®^ Fixable Near IR dye (Invitrogen) and anti-mCD4-PE (**Table S2**), then analyzed for the proliferation profile of the CellTrace Violet labeled responder population *via* flow cytometry. Data were acquired using BD LSRII or FACSCelesta analyzer with proper compensation settings and gating (**Figure S5**). Frequencies of proliferating responder cells were quantified using FCS Express 6.05 software. To compare the suppressive capacity of iTreg of different sources, non-linear inhibition modeling was performed using Prism GraphPad v8.4.3 software, with IC50 (half-maximal inhibitory iTreg concentration) reported.

### Islet Isolation

All animal procedures were conducted under IACUC approved protocols at the University of Florida, Gainesville, FL. Islets were isolated from Lewis rats or Balb/C mice, as previously described (36). Briefly, pancreatic tissue is digested by injecting collagenase (Liberase, Roche) *via* cannulation of the bile duct. Islets were then separated from acinar cells and pancreatic tissue *via* density gradient separation (Ficoll). Isolated islets were maintained in complete media (CMRL 1066 media (Mediatech, Inc.) supplemented with 10% FBS (Hyclone Laboratories Inc, Cytiva), 20 mM HEPES buffer, 100 U/mL penicillin‐streptomycin, and 2 mM L‐glutamine) for 48 hours before transplantation.

### *In Vitro* Coculture of Islets and TGF-β1 PLGA Microparticles

The cytocompatibility of TGF-β1 PLGA MPs was evaluated by co-incubating 500 IEQ Lewis rat islets with 250 μg TGF-β1/PLGA MPs or 3 ng/mL soluble TGF-β1 in a 24-well transwell insert in 1 mL of complete CMRL media for 48 hours under standard culture conditions (5% CO_2_, 37°C). Rat islets were used for *in vitro* screening due to their higher islet yield per donor. The viability of islets was assessed *via* both Live/Dead^®^ confocal imaging and MTT assay. The function of islets was assessed using glucose-stimulated-insulin-release (GSIR) assay.

Islet function was evaluated *via* GSIR assay. Briefly, 150 IEQ islet post coincubation were immobilized in chromatography columns using Sephadex G10 resin beads (Cytiva), followed by sequential stimulations with 3 mM (Low 1), 11 mM (High), and lastly another 3 mM glucose (Low 2) for one hour for each step respectively. Eluant samples (1 mL) collected after each one-hour stimulation were analyzed for insulin content *via* ELISA (Mercodia Inc.) and normalized by PicoGreen DNA content (Invitrogen), as previously published (36, 37).

For Live/Dead imaging, islets post coincubation were stained with 26.67 μM calcein AM and ethidium homodimer-1 (Invitrogen) in PBS at 37°C for 30 min, followed by confocal imaging (Zeiss LSM 710). Islets were maintained in complete media during image acquisition.

For the MTT assay, islet metabolic activity was assessed following the manufacturer’s instruction (Promega). Briefly, 250 IEQ islets post co-culture was incubated in 250 µL complete media with 28 µL MTT reagent for 1 hour. Then the reaction was terminated by stop solution (185 µL), followed by a 48-hour formazan crystals solubilization and signal development. Absorption of samples was read at 570 nm using a spectrophotometer (Molecular Devices, LLC).

### Allogeneic Islet Transplantation

For islet recipients, diabetes was induced in male C57BL/6 mice *via* intravenous injection of streptozotocin (STZ) (200 mg/kg, Sigma-Aldrich), with hyperglycemia confirmed by three or more consecutive days of non-fasting blood glucose levels above 300 mg/dL, as previously described (38). This chemical induction protocol has consistently resulted the generation of a durable diabetic state, as validated through extensive survival graft retrievals using this animal model (38–40). Allogeneic islets were sourced from Balb/c mice donors as mentioned above. Epididymal fat pads (EFPs) were used as the transplant sites, as previously described (36, 41). In brief, a dosage of 1,000 IEQ/recipient (500 IEQ/EFP) was placed into the spread EFP using a Hamilton glass syringe, in accordance with previously published reports (42, 43). For mice receiving PLGA TGF-β1 microparticles, 10 mg of particles were then placed within the transplant site using the same sterile Hamilton glass syringe. After delivery of the islets or islets+MPs, fibrin glue was applied on top of the implanted material to hold in place. The tissue was then wrapped and the EFP was sealed using additional fibrin glue. Tested groups included allogeneic islets alone (8 recipients, n=8) and allogeneic islets with PLGA TGF-β1 MPs (10 mg MPs per EFP, 14 recipients, n=14).

### Graft Function Monitor and Graft Retrieval

Post-transplantation, the non-fasting blood glucose levels and weights of the animals were monitored until graft rejection. Mice with three consecutive readings of non-fasting blood glucose below 200 mg/dL were classified as normoglycemic, indicating engraftment. Graft rejection was considered when three or more consecutive readings of non-fasting blood glucose above 200 mg/dL were observed.

When rejection occurred, islet grafts, spleens, and lymph nodes (brachial, inguinal, and mesenteric LNs) of the rejecting recipients were harvested for downstream characterization. Mice maintaining normoglycemia for more than 90 days were classified as non-rejecting (long-term engraftment), then euthanized for graft and tissue retrieval as mentioned above.

### Intraperitoneal Glucose Tolerance Test (IPGTT)

For mice maintaining normoglycemia for more than 60 days, IPGTT was performed between 60-70 days post-transplant to assess the function of islet grafts. Briefly, mice were fasted overnight and given an intraperitoneal injection of 20% (w/v) Dextrose at a dose of 1/100 body weight. Naïve age-match mice (n=3, 20 week-old) were included as an additional control. Blood glucose was then monitored over 90 mins, or until normoglycemia was achieved.

### Histology

Explants were fixed in 10% formalin, followed by paraffin embedding and sectioning (10 μm). Sections were stained with hematoxylin and eosin (H&E) and Masson’s trichrome stain following manufacturer’s protocol, then imaged using a light microscope at 20x magnification (Zeiss Axio Observer).

For immunohistochemistry, tissue sections were de-paraffinized, antigen-retrieved (120°C for 20 min in citrate buffer), and stained with anti-CD3, anti-Foxp3 and anti-insulin primary antibodies for marker labeling (**Table S2**). Secondary antibodies with fluorophores of AlexaFluor 568, AlexaFluor 647, and AlexaFluor 488 were then applied for signal generation (**Table S2**). Nuclei were stained with DAPI for 1 hour at RT (1:5000; Life Technologies). Immunofluorescent staining was imaged by Zeiss LSM 710 or Leica TCS SP8 confocal microscope with isotype control to ensure signal specificity. Signal quantification and cell density (DAPI area) of each sample was quantified following the previously published protocol using ImageJ software (44). CD3 and Foxp3 signals were quantified as the area of CD3^+^ or Foxp3^+^ staining normalized by the DAPI^+^ area (cell containing tissue area) in each image, with five independent images analyzed per group. To characterize host CD3^+^ cell infiltration into the islet, CD3 signal within the defined islet area was quantified and normalized to the total DAPI^+^ area.

### Mixed Lymphocyte Reaction (MLR)

Splenocytes of all allogeneic islet recipients were collected and used in MLR assays to characterize systemic immunotolerance, as previously reported (45). Briefly, splenocytes of recipient mice were labeled with CellTrace Violet dye and co-cultured with mitomycin c treated splenocytes from a naïve C57BL/6 mouse (syngeneic), Balb/c mouse (allogeneic), or C3H mouse (third-party stimulators) at a 1:1 ratio. MLR responses were quantified by flow cytometry staining for the proliferation of CD8^+^ cells after five days. Immune responses of the splenocytes sourced from a 12 to 15 week-old naïve C57B/6 mouse were used as controls.

### CD4^+^ T Cell Immunophenotyping

Brachial, inguinal and mesenteric LNs, and spleens were collected from allogeneic islet recipients exhibiting long-term efficacy (e.g. > 90 days post-transplantation). Procured lymphocytes were stained for flow cytometric analysis of CD4^+^ T cell phenotype fresh without re-stimulation. Cells were stained with anti-mCD4-AF700, anti-mCD8-PE, anti-mFoxp3-AF488, anti-mTbet-Pacific Blue, anti-mGata3-PerCpCy5.5, and anti-mRorγt-PE610 (**Table S2**). Samples were analyzed on an LSRII flow cytometer (BD), with data analysis performed using FCS Express 6.05 software (De Novo Software).

### Statistical Analysis

The power of tests and the statistical methods are described throughout the article and in the figure legends. Generally, statistical assessments were performed using one-way ANOVA with Tukey’s multiple comparison analysis using GraphPad Prism 8.4.3 software. Statistical difference is considered significant when the probability value (*p*) is <0.05. Difference was shown as *p<0.05; **p<0.01; ***p<0.001; **** p < 0.0001 and *n.s.* indicates *not significant*.

## Results

### Fabrication and Characterization of TGF-β1 PLGA MPs

**Figure 1:**
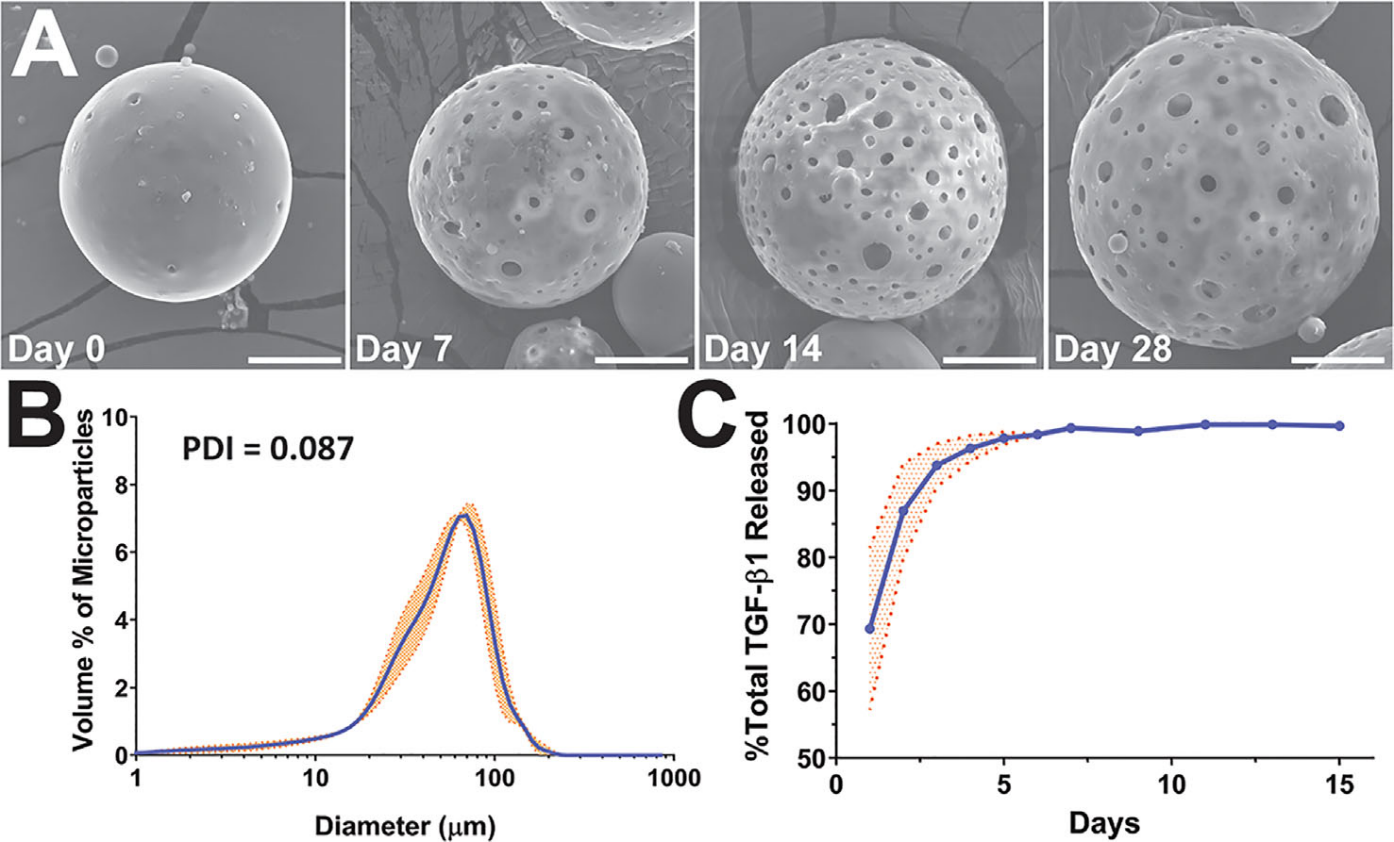
Characterization of TGF-β1 PLGA microparticles (MPs). **(A)** Representative SEM images of the surface morphology of TGF-β1 PLGA MPs along with the releasing studies (collection time noted). Tests were performed twice independently with n = 5 per time point. Scale bar = 15 μm. **(B)** Size distribution and the calculated polydispersity index (PDI) of TGF-β1 PLGA MPs, as determined by laser diffraction. Data is the mean of three independent fabrication batches, measured in triplicates (N=3, n=9, blue line) with standard deviation (orange shade). **(C)** Release profile of TGF-β1 PLGA microparticles normalized to total protein release. Mean TGF-β1 release curve (blue line) was acquired by averaging four independent (N=4, n=13) studies with particles of different batches, with standard deviation (orange shade) as shown.

PLGA microparticles encapsulating TGF-β1 were fabricated using a double emulsion method, with formulation A (**Table S1**) serving as the baseline formulation (46). The resulting TGF-β1 PLGA MPs were spherical (Day 0, **Figure 1A**) with a desired size range and monodistribution (PDI = 0.087, N=3) that minimized APC phagocytosis while also supporting injectability and ease in co-implantation with islets (mean diameter of 54 ± 51 µm; **Figure 1B**) (20).

The encapsulation efficiency of TGF-β1 in PLGA MPs was 49.0 ± 0.1%; a percentage in-line with published reports (16, 47–49). TGF-β1/PLGA MPs were hydrated for time-course drug release profiling and SEM imaging. Following hydration, the MPs became more porous and swollen, as shown in **Figure 1A**, indicating active PLGA degradation and TGF-β1 release over time. The kinetic release profile of TGF-β1 PLGA MPs exhibited a burst release of 69.33 ± 12.12% after 24 hr, with 95% of the total release occurring after five days (**Figure 1C**). To avoid inadvertent immune cell activation, endotoxin levels of TGF-β1/PLGA MPs (10 mg/mL) were measured, yielding 0.15 ± 0.01 EU/mL *via* LAL assay, which is below the FDA design criteria of <0.5 EU/mL for biomaterial devices (**Table S3**) (50). Different batches of TGF-β1/PLGA MPs (N > 3) produced similar results, indicating the stability and reproducibility of the particle fabrication process.

Given that the duration of TGF-β1 release within the local graft site may play a role in the global regulatory environment and subsequent allograft protection, we sought to potentially extend TGF-β1 release kinetics using a concurrent iterative design approach. Protein release from PLGA microparticles typically occurs in phases: first, a diffusion dependent burst release; followed by a lag phase; and, depending on the PLGA properties and the protein entrapment, a second release phase controlled by polymer degradation in which deeply entrapped protein is released (51). Most publications using TGF-β1/PLGA particles have reported a diffusion dependent release, similar to the profile shown in **Figure 1C**, with minimal additional release occurring after the initial burst (16, 47–49). Examination of PLGA literature for other agents, however, indicates the potential for modified kinetics. For example, a higher lactic acid to glycolic acid ratio PLGA may induce a longer release profile, as an elevated degradation rate can permit the release of proteins more deeply entrapped within the particle. Decreasing the molecular weight and viscosity of the PLGA is another approach, whereby increasing the compactness of the particle can subsequently slow the entrapped protein release (51). Finally, adding agents to increase the osmotic pressure in the particle collecting fluid can drive water away from the inside of the particle and result in more deeply entrapped proteins (52). To explore the feasibility of these modifications, additional TGF-β1/PLGA MPs were generated with manipulation of polymer degradation *via* lactic acid:glycolic acid ratio (formulations B, D), particle compactness *via* polymer viscosity (formulations B, C, and D), and aqueous phase entrapment *via* the addition of an osmotic agent (formulation E), as summarized in **Table S1**. It was hypothesized that these changes would serve to both dampen early burst levels and extend the release of TGF-β1.

The MP design alterations did not significantly change the overall particle mean diameter (*p = 0.47*; one-way ANOVA; **Figure 1**); however, the lower viscosity polymer formulations did exhibit an increased contribution of smaller particles (**Figures S1B–D**). Surprisingly, none of the formulation modifications significantly altered TGF-β1 release kinetics (**Figure S2**). In addition, no significant differences in encapsulation efficiency were observed for the new formulations, when compared to the baseline formulation A (**Figure S2**). Due to the lack of alterations in TGF-β1 release properties, the baseline formulation A was used for all subsequent *in vitro* and *in vivo* experiments.

### TGF-β1 PLGA MPs Generate Polyclonal and Antigen-Specific iTregs *In Vitro*

**Figure 2:**
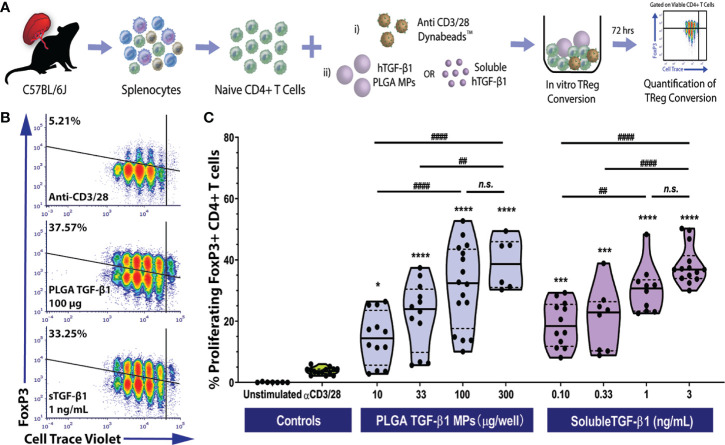
Efficient Polyclonal Foxp3^+^ iTreg Conversion by PLGA Microparticles Releasing TGF-β1 *In Vitro*. **(A)** Schematic of polyclonal iTreg conversion assay. Conversion rate was assessed by flow cytometry analysis of Foxp3 expression after the three-day co-culture of 10^5^ magnetically sorted splenic naïve C57BL/6 CD4^+^ T cells with anti-CD3/CD28 activator beads and TGF-β1, within either PLGA MPs or soluble format. **(B)** Representative flow cytometric density plots (gated on viable CD4^+^ T cells) showing the frequency of Foxp3^+^ induced Tregs resulting from polyclonal stimulation (anti-CD3/28) in the presence of PLGA TGF-β1 MPs or soluble TGF-β1. **(C)** Summary of polyclonal iTReg generation, characterized as the proliferating CD4^+^Foxp3^+^ cells (refer to **Figure S3**), following incubation with the designated agents. Data were shown in a truncated violin plot with the mean (solid black line) and individual data points (N=4; n=16). Paired Tukey’s test was conducted for mean comparison, with * used when compared to control group (activator beads only) and # for comparison within TGF-β1 groups. Statistical significance was determined as **** or ^####^p < 0.0001, ***p < 0.001, ** or ^##^p < 0.01, *p < 0.05 and n.s., not significant.

The bioactivity and immunoregulatory effects of our PLGA/TGF-β1 MPs were tested *via* an *in vitro* co-culture assay. Specifically, the capacity of these particles to convert naïve CD4^+^ T cells into regulatory CD4^+^Foxp3^+^ T cells (iTregs) was quantified. For this T cell conversion, purified naïve CD4^+^ T cells (i.e., CD4^+^CD44^low^CD62L^high^) from C57BL/6 mice were polyclonally activated by anti-CD3/CD28 Dynabeads in the absence or presence of PLGA TGF-β1 MPs, followed by the downstream measurement of iTreg cell generation (**Figure 2A**). Four TGF-β1 MP dosages were tested: 300, 100, 33, and 10 µg of MPs per well with 10^5^ naïve CD4^+^ T cells. An experimental group treated with soluble TGF-β1 was also screened. The selection of soluble TGF-β1 dosages (3, 1, 0.33 and 0.10 µg TGF-β1 per well with 10^5^ naïve CD4^+^ T cells) was based on both the theoretical and experimental TGF-β1 release from the microparticles (summarized in **Table S4**), thus permitting a comparison in conversion efficiency between soluble and PLGA-released TGF-β1. To ensure the resulting Foxp3^+^ CD4^+^ T cells detected at the experimental endpoint were TGF-β1-induced iTregs, a highly efficient naïve CD4 T cell isolation kit was employed in this study and the purity of the resulting naïve CD4^+^ T cells was validated by cytometric assay. Greater than 95% purity of naïve CD4^+^CD62L^+^ was measured in the purified population (**Figure S6**). Importantly, a minimal contribution of CD4^+^Foxp3^+^ cells was detected in the naïve CD4^+^ population, within which low levels of both helios^+^ (1%) and helios^-^ (1.25%) subpopulations were measured. The highly purified final naïve CD4^+^ population also exhibited a high ratio of Foxp3^-^helios^-^ cells to Foxp3^+^helios^-^ cells (**Figure S6D**).

As summarized in **Figures 2B, C**, the PLGA TGF-β1 MPs effectively generated polyclonal iTregs in a dose-dependent manner. While controls containing only anti-CD3/28 activator beads showed a modest frequency of viable Foxp3^+^ CD4^+^ T cells (3.73± 1.12%), the presence of PLGA TGF-β1 MPs, even at a low dose (10 µg per well of 10^5^ CD4^+^ T cells), resulted in a significant increase of iTreg generation (*p = 0.02*, Tukey post-hoc). Increasing levels of iTregs were observed as the dose of PLGA/TGF-β1 MPs increased up to 100 µg TGF-β1 PLGA MPs per reaction (10^5^ CD4^+^ T cells). At the higher doses of 300 µg TGF-β1/PLGA MPs, a plateau in TGF-β1-stimulated iTreg generation was reached, with a peak conversion rate ~39% and no significant change from the 100 µg MP dosage (**Figure 2C**). Similar to the iTreg conversion by PLGA MPs, efficient Treg conversion was observed by soluble TGF-β1, with about 19.05 ± 7.3% conversion detected with the lowest dose of tested (0.1 ng/mL). A positive correlation between iTreg frequency and soluble TGF-β1 dosage was also observed, with a plateau after the 1 ng/mL dosage (**Figure 2C**). An overall comparison of TGF-β1 experimental groups found that dosage (p < 0.001), but not delivery method (soluble or PLGA-releasing; *p = 0.69*), significantly impacted polyclonal iTreg generation (two-way ANOVA). The observed Treg conversion from TGF-β1-releasing PLGA MP was also specific to TGF-β1 and not induced by the PLGA material, as BSA-releasing PLGA control particles did not promote iTreg generation, when compared to untreated controls (**Figure S8**). Collectively, these results showed the TGF-β1/PLGA MPs were capable of releasing bioactive TGF-β1 molecules and converting naïve T cells into the immunomodulatory Foxp3^+^CD25^+^ iTregs in a manner comparable to soluble TGF-β1.

**Figure 3:**
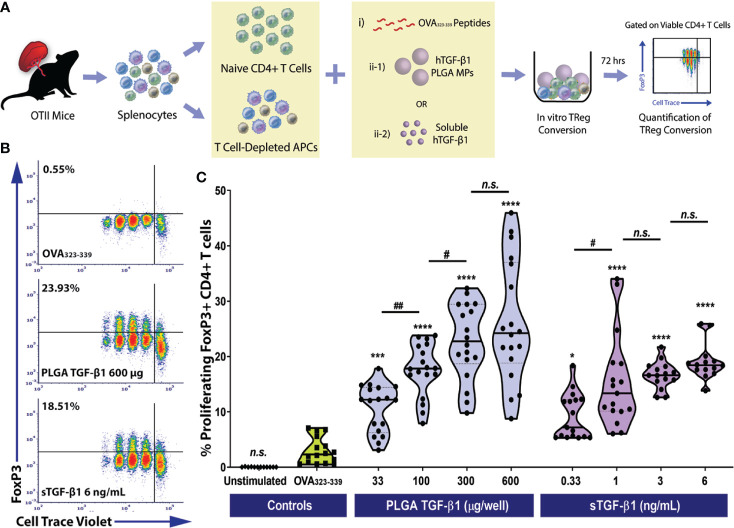
Efficient Monoclonal Foxp3^+^ iTreg Conversion by PLGA Microparticles Releasing TGF-β1 *In Vitro*. **(A)** Schematic of the antigen-specific Foxp3^+^ iTreg generation assay using PLGA TGF-β1 MPs. Naïve OTII CD4^+^ T cells were stimulated using 0.5 μM OVA_323-339_ peptide and mitomycin C treated syngeneic APCs, along with immunomodulation by either PLGA TGF-β1 microparticles or soluble TGF-β1. The frequency of proliferating Foxp3^+^ CD4^+^ T cells was quantified by flow cytometry after three days. **(B)** Representative cytometric density plots of Foxp3^+^ iTreg (noted in upper left quadrant, gated on viable CD4^+^ T cells) following co-incubation with OVA_323-339_ peptide, APCs, and either PLGA TGF-β1 MPs or soluble TGF-β1. **(C)** Summary of antigen-specific iTreg generation using PLGA TGF-β1 MPs. Data were shown in a truncated violin plot with the mean (solid lines) and individual data points (N=4; n=18). Outliers were identified and excluded using ROUT method with multiplier Q=1%. Paired Tukey’s test was conducted for mean comparison, with * is used when compared to control group (OVA_323-339_ peptide and APCs only) and # for comparison within TGF-β1 groups. Statistical significance was determined as ****p < 0.0001, ***p<0.001, ^##^p<0.01, * or ^#^ represents p < 0.05 and n.s., not significant.

As T1D pathogenesis is thought to be self-antigen driven (53), the ability to generate antigen-specific Tregs may prove advantageous for islet graft acceptance, especially for recipients with established autoimmune memory (54). To investigate the ability of TGF-β1-releasing PLGA MP to generate antigen-specific iTregs, antigen specificity was employed using an ovalbumin (OVA) specific OTII CD4^+^ T cell model (55). Specifically, naïve OTII CD4^+^ T cells were stimulated by OVA_323-339_ peptide, presented by syngeneic APCs, and co-cultured with either particle-releasing or soluble TGF-β1 of titrated dosages (33-600 µg/10^5^ naïve CD4^+^ T cells for TGF-β1/PLGA MPs and 0.33-6 ng/mL for soluble TGF-β1) (**Figure 3A**).

Similar to polyclonal conversion, in a three-day timeframe, efficient antigen-specific OTII CD4^+^ iTregs generation was observed using TGF-β1/PLGA MPs, as shown in the representative cytometric density plots (**Figure 3B**). For example, the frequency of iTregs resulted from the treatment using 300 µg TGF-β1/PLGA MPs was 22.76 ± 11.02%, which was over 7.7-fold higher than the controls (OVA_323-339_ only, 2.95 ± 2.37%, *p<0.0001*, Tukey post-hoc) and equivalent to the monoclonal iTreg level induced by the corresponsive 3 ng/mL soluble TGF-β1 dose (17.62 ± 4.66%, *p=0.13*, Tukey post-hoc). In addition, a dose-dependency of TGF-β1/PLGA MPs was shown in OVA-specific iTregs induction, with a plateau observed at 600 µg TGF-β1/PLGA MPs per 10^5 naïve CD4^+^ T cells (**Figure 3C**), equivalent to treatment with 6 ng/mL soluble TGF-β1 (*p=0.50*, Tukey post-hoc). To validate the specificity of iTreg conversion, cytometric quantification of helios expression was performed on the OTII CD4^+^ T cells following *in vitro* conversion. As shown in **Figure S7C**, a low level of helios^+^CD4^+^Foxp3^+^ cells was detected, ranging from 0.47-2.8% of the total viable CD4^+^ T cells, depending on the dose of TGF-β1 applied (**Figure S7C**). Moreover, as the percentage of iTreg (Foxp3^+^helios^-^CD4^+^ cells) significantly increased (**Figure S7B**), there was a corresponding decrease in naïve Foxp3^-^helios^-^CD4^+^ T cells (**Figure S7A**), supporting the generation of iTregs from the naïve CD4 T cell pool *via* TGF-β1/PLGA MPs or sTGF-β1. Collectively, these results found TGF-β1/PLGA MPs are capable of generating antigen-specific CD4^+^Foxp3^+^ iTregs *in vitro* with similar efficacy as soluble TGF-β1.

### iTregs Induced by TGF-β1 PLGA Microparticles Are Functionally Suppressive

Transient Foxp3 expression and non-function for *in vitro* induced human Tregs has been previously reported (56). Thus, it was important to establish that the iTregs generated by TGF-β1/PLGA MPs were functionally suppressive. The functional potency of the Tregs was validated by tracking the activation and proliferation of the CD4^+^ responder T cell population in a co-culture system with iTregs (35). The ratio of T regulatory cells to T responders (Treg : Tconv) was also varied to characterize dose effects. For this study, T regulatory cells of three sources were tested: natural Treg (nTreg; endogenous CD4^+^Foxp3^+^ T cells), TGF-β1/PLGA MPs iTregs, and soluble TGF-β1 iTregs. For this study, a transgenic Treg reporter mouse was used, C57BL/6-FIR or FoxP3^RFP^. As only RFP^-^ cells were used to generate iTregs, this study served as an additional validation that TGF-β1 induced Treg generation from naïve CD4^+^ T cells.

**Figure 4:**
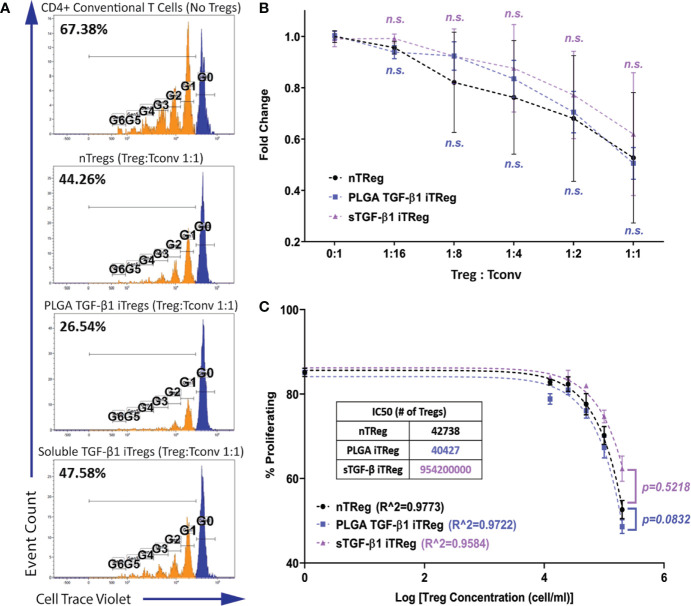
iTregs Induced by TGF-β1 PLGA Microparticles are Functionally Suppressive. Freshly isolated natural Tregs (nTregs), or iTregs generated by either TGF-β1 PLGA MPs or soluble TGF-β1 were mixed with CellTrace labeled CD4^+^Foxp3^−^T responder cells and stimulated with anti-CD3 in the presence of syngeneic APCs. Five different Tregs: Tconv ratios were tested (1:16, 1:8, 1:4, 1:2, and 1:1). **(A**) Representative responder cell proliferation histograms with the highest Treg dose (1:1 Treg : Tconv ratio) compared to control with no suppression. **(B)** Summary of the suppression of CD4^+^ responder T cell proliferation, as characterized by the percentage of dividing population normalized to the control with no suppression. N = 3, n = 10. n.s., not significant *via* two-way ANOVA test. **(C)** Non-linear suppression modeling of iTreg suppression of a representative test, where R^2 is the goodness-of-fit and IC50 is the half-maximal inhibitory concentration (inset).

As shown in **Figure 4**, potent suppression of the responder cell proliferation was observed when Tregs were added to the system. Regardless of the Treg source, the degree of responder suppression was concentration-dependent, with the highest Treg ratio (1:1) imparting the highest suppression of responder T cell proliferation (**Figure 4B**). The immunosuppressive function of PLGA/TGF-β1 iTregs was insignificantly different to nTregs for all doses tested (**Figure 4B**). Further analysis using non-linear inhibition modeling also showed similar suppressive functions of PLGA/TGF-β1 iTregs and nTregs (*p=0.08*, **Figure 4C**). T regulatory cells generated by soluble TGF-β1 showed similar suppressive capabilities to nTregs, both in responder suppression and inhibition modeling (**Figures 4B, C**). Collectively, these results support that PLGA/TGF-β1 iTregs were functionally suppressive with efficacy comparable to natural Tregs.

### Islet Cytocompatibility of TGF-β1 PLGA Microparticles *In Vitro*

**Figure 5:**
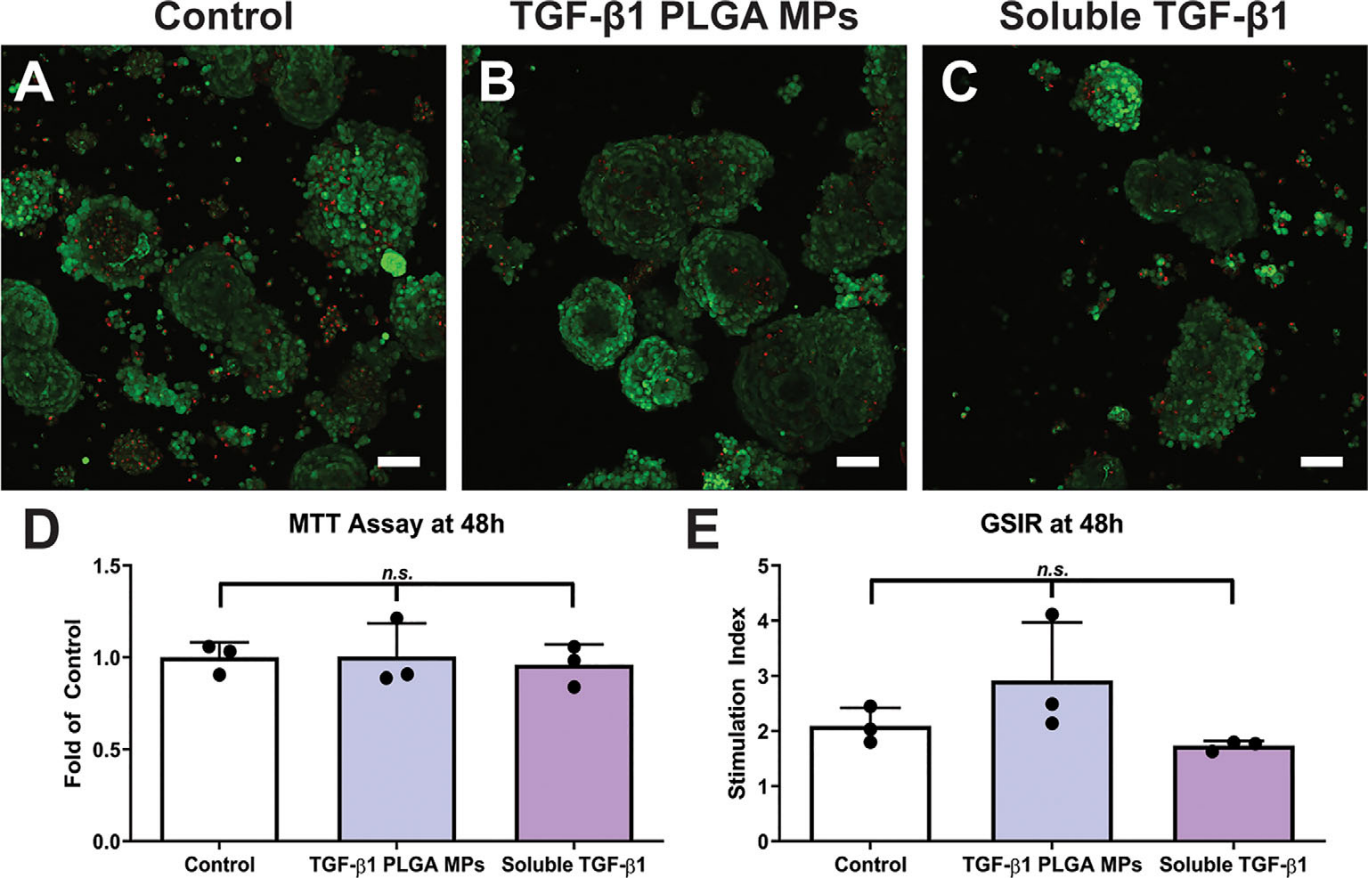
Cytocompatibility of TGF-β1 PLGA Microparticles with Rat Pancreatic Islets. Representative LIVE/DEAD images of **(A)** control islets, **(B)** islets incubated with TGF-β1 PLGA microparticles, and **(C)** islets incubated with soluble TGF-β1. Red = dead cells (EthD-1), Green = viable cells (Calcein AM). Scale bars = 50 µm. **(D)** MTT metabolic assay, n=3. **(E)** Stimulation index resulted from glucose stimulated insulin response, High/Low 1, n=3. Paired Tukey’s test was conducted for mean comparison, with n.s., not significant.

With the vision of co-transplanting TGF-β1 PLGA MPs with pancreatic islets to modulate the local graft microenvironment and promote graft acceptance, the cytocompatibility of these particles with islets was evaluated *in vitro*. The incubation of murine pancreatic islets with TGF-β1/PLGA MPs or soluble TGF-β1 imparted no significant difference in overall visual viability, global metabolic activity, or glucose-sensing insulin-secretory function, as summarized in **Figure 5**. Thus, these results indicate islet compatibility of local TGF-β1 release *via* PLGA MPs.

### Allogeneic Islet Transplantation With TGF-β1 PLGA Microparticles

**Figure 6:**
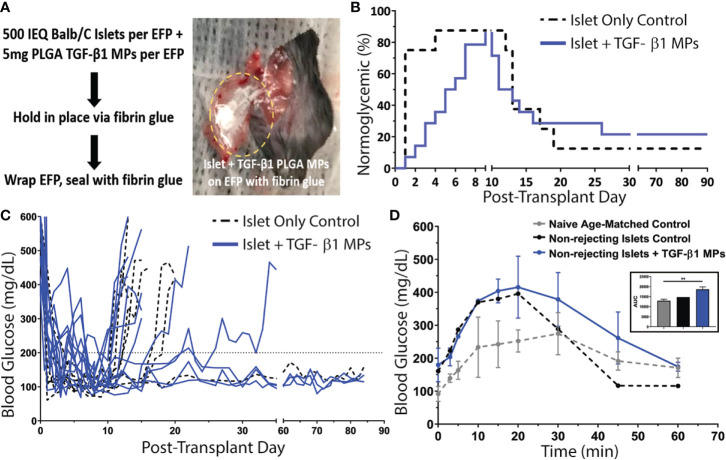
Impact of TGF-β1 PLGA Microparticles on the Efficacy of Allogeneic Islet Transplant in Diabetic Murine Model. **(A)** Balb/c islets were co-transplanted with or without TGF-β1 PLGA MPs in the EFPs of diabetic C57BL/6 recipients, followed by the immobilization and seal with fibrin glue. **(B)** Survival curves on normoglycemia (BG < 200 mg/dL) for allogeneic islets recipients with (blue line, n=14) or without (black line, n=8) TGF-β1 PLGA MPs (blue line, n=14) post-treatment. Graft rejection was defined as three consecutive BG readings ≥ 200 mg/dL. **(C)** Blood glucose level for individual graft recipients for control (black, n=7) and TGF-β1 PLGA MPs (blue, n=12) groups, with PNF recipients excluded. **(D)** Intraperitoneal Glucose Tolerance Test (IPGTT) of mice with long-term functioning allogenic islet grafts performed between 60-70d post-transplant, with n=3 for naïve age-matched control (grey), n=1 for control (black) and n=3 for TGF-β1 PLGA MPs (blue). The area under curve (AUC) was quantified and shown in the inset.

Following promising *in vitro* validation, PLGA TGF-β1 MPs were incorporated into an extrahepatic murine allogeneic islet transplant model. The epididymal fat pad (EFP) was used as the transplant site, as it is a favorable extrahepatic location for murine islet transplantation and analogous to clinically relevant sites such as the omentum (36, 40, 57). A dosage of 1,000 IEQ allogeneic Balb/c islets (500 IEQ per EFP) was transplanted into full MHC mismatched chemically-induced diabetic C57BL/6 recipients (**Figure 6A**). Two groups were examined in this study: standard allogeneic islet-only controls (n = 8); and allogeneic islets co-transplanted with TGF-β1 PLGA MPs (n = 14; 10 mg MPs/recipient with 5 mg MPs per EFP). A 10 mg of TGF-β1 MPs per recipient (5mg per EFP) dosage was selected to balance both the known rapid degradation and clearance of TGF-β1 *in vivo* (58) while also decreasing the risk of potential off-target immune impacts and deleterious fibrosis (59, 60). A PLGA-only group was not included in the transplant study, given that *in vitro* screenings did not indicate a benefit of the material in iTreg induction (**Figure S8**) and the documented minimal immunomodulatory effects and allograft protection reported using PLGA-only vehicles (17, 61–63).

The average time to achieve normoglycemia post-transplantation was 1 ± 1 day(s) for control animals and 5 ± 2 days for TGF-β1/PLGA MPs treated mice (**Figure 6B**), with no significant difference in the reversal time between these two groups (*p = 0.10*, Mantel-Cox log-rank test). As summarized in **Figure 6B**, one control (12.5%) and two TGF-β1 PLGA MPs treated mice (14.3%) exhibited primary non-function (PNF) and were excluded from subsequent characterization. For successfully engrafted islet recipients, after the brief normoglycemic period, 6 of the 7 allogeneic islet-only controls rejected (86%) with an average rejection time of 15 ± 3 days post-transplantation. Meanwhile, 9 of the 12 allogeneic islet grafts treated with TGF-β1 PLGA MPs rejected (75%) with rejecting grafts destabilizing on average 14 ± 5 days post-transplantation. Collectively, no significant difference in rejection rates was measured between the control and TGF-β1 PLGA MPs treated groups (Mantel-Cox log-rank; *p = 0.97*, **Figure 6B**). Examination of non-fasting blood glucose levels (**Figure 6C**) also revealed no global impact of the local TGF-β1/PLGA MPs on glycemic control of functional grafts.

The functional response of the engrafted islets in recipients who showed stable graft function after 60 days post-transplant was also measured *via* dynamic glycemic challenge (IPGTT). Although, the glucose tolerance was lower compared to age-matched non-diabetic naïve mice, glucose clearance of both control (n=1) and TGF-β1 PLGA particle treated (n=3) groups was efficient, with a return to normoglycemia (<200 mg/dL) within 60 min (**Figure 6D**) and equivalent glucose clearance (p = 0.068, one-way ANOVA, classified as the area under the curve) (**Figure 6D**, inset).

**Figure 7:**
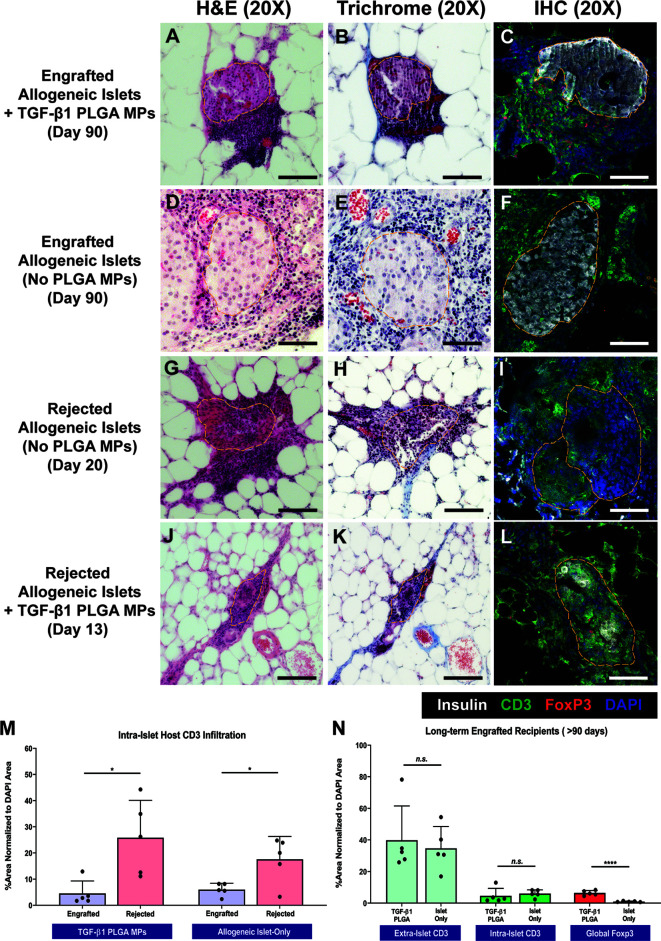
Representative Histological Images of Islet Graft Retrieved in Allogeneic Islet Transplantation. Representative H&E staining, Masson’s Trichrome staining, and immunohistochemistry staining for insulin (white), CD3 (green), Foxp3 (red), and DAPI (blue) on tissue from non-rejecting islet graft with TGF-β1 PLGA MPs **(A–C)**, non-rejecting allogeneic islet graft-only **(D–F)**, rejected allogeneic islet grafts **(G–I)**, and rejected islet grafts with TGF-β1 PLGA MPs **(J–L)**. Scale bars = 100 µm. Quantification of **(M)** intra-islet CD3^+^ cell infiltration for both engrafted and rejected allogeneic islet grafts, normalized to the total area of DAPI. **(N)** Quantification of intra-islet CD3^+^ cells, extra-islet CD3^+^ cells and global Foxp3^+^ cells normalized to the total area of DAPI were summarized for long-term engrafts recipients. t-test was performed between engrafted versus rejected islets, or TGF-β1 PLGA MPs treated and islet-only groups. n=5. ****p < 0.0001, *p < 0.05 and n.s., not significant.

Grafts from both rejecting and long-term surviving (> 90 days) recipients were explanted for histological characterization *via* H&E, trichrome, and immunohistochemical staining. For long-term functional recipients who were treated with TGF-β1/PLGA MPs (>90 days), H&E and trichrome staining (**Figures 7A, B**) revealed intact and re-vascularized islets, with a moderate accumulation of nucleated host cells adjacent to, but not migrating into, the islets. IHC staining validated these trends, with robust insulin staining within islets and CD3^+^ T cells residing at the periphery of the islet graft (**Figure 7C**). As a comparison, long-term engrafted allogeneic islet grafts without particle treatment also showed intact islet morphology and integrity (**Figures 7D, E**), with host T cell accumulation but major indicators of active T cell invasion (**Figure 7F**).

In contrast to the functional grafts, control allogeneic islets rejected after 14-20 days post-transplant exhibited extensive host T cell infiltration and enhanced collagen deposition with little islet tissue/structure remaining (**Figures 7G–I**), implying host-mediated graft destruction. Rejected grafts that received TGF-β1/PLGA MPs were also examined histologically (**Figures 7J–L**), with retrieval dates dependent on the rejection time (i.e., 13-35 days post-transplant). Similar to control rejected grafts, notably fewer islets were observed, with fragmented but discernable morphology and minimal insulin signal (**Figures 7J–L**).

Image quantification of grafts validated these observations and also revealed immunomodulatory impacts of the TGF-β1/PLGA MPs treatment. Specifically, the comparison of rejected versus nonrejected islets for the same treatment group measured a significant increase in intra-islet CD3^+^ T cells infiltration (p = 0.013 and 0.002 for control and TGF-β1/PLGA MPs treated groups, respectively; **Figure 7M**), supporting T cell-mediated islet graft rejection. Focusing on long-term engrafted implants, the incidence and infiltration of T cells into the islets were equivalent for both control and TGF-β1/PLGA MPs treated implants (p = 0.67 and 0.56 for extra-islet and intra-islet CD3^+^ T cells, t-test; **Figure 7N**). Investigation into the presence of T regulatory cells, however, indicated a local regulatory effect of the TGF-β1/PLGA MPs. Long-term islet grafts treated with TGF-β1/PLGA MPs exhibited higher levels of Foxp3^+^ CD3^+^ T cells, when compared to control, but functional, grafts (6.41 ± 5.13% versus 0.96 ± 0.32% global Foxp3^+^ cell infiltration; p < 0.0001, t-test; **Figure 7N**). Of additional interest, rejected grafts containing TGF-β1/PLGA MPs also exhibited a regulatory microenvironment, with elevated Foxp3^+^ cell infiltration when compared to rejected explants with no particle treatment (p = 0.005, t-test) and at a level similar to explants from long-term functioning grafts containing TGF-β1/PLGA MPs (5.17 ± 2.97%; p = 0.43, t-test). These results indicate that local TGF-β1 delivery is imparting a local T cell regulatory effect, despite rejection of 75% of the functional grafts.

### Long-Term Allogeneic Islet Graft Tolerance Achieved With TGF-β1 PLGA MPs Localized Immunomodulation

To provide further insight into the impacts of TGF-β1/PLGA MPs on the immune system of the recipients, CD4^+^ T cells harvested from lymphoid organs (lymph nodes and spleens) of particle-treated non-rejecting allogeneic islet recipients were immunophenotyped. Specifically, T cells procured from spleens, proximal (mesenteric and inguinal), and peripheral (brachial) lymph nodes (LN) were examined for Treg (Foxp3^+^), Th1 (Tbet^+^), Th2 (Gata3^+^), and Th17 (RoRγt^+^) lineages. Of note, to properly capture the CD4^+^ T cell polarization of these recipients, no re-stimulation was applied to the CD4^+^ T cells prior to the phenotyping.

**Figure 8:**
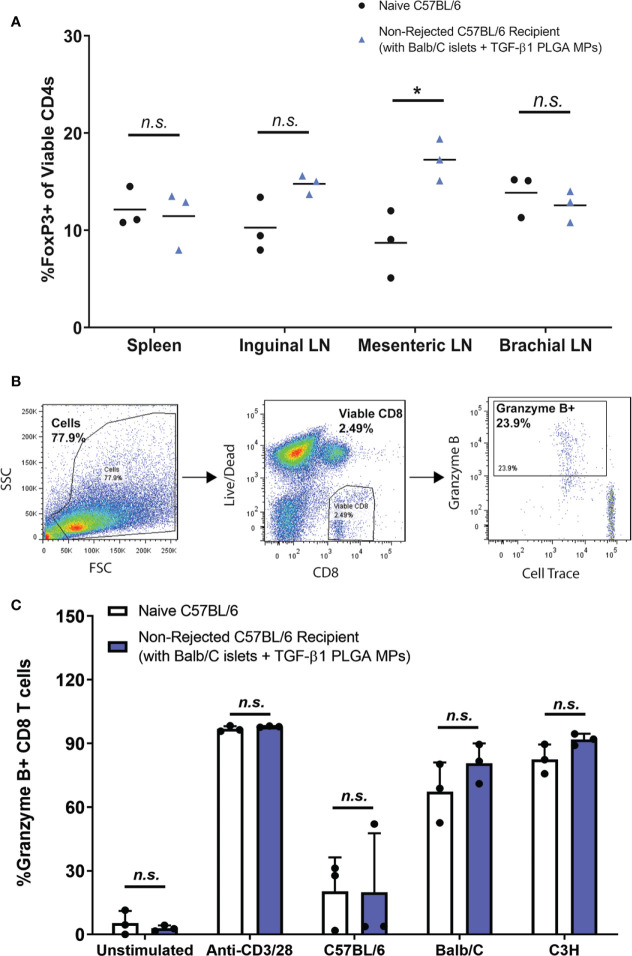
Immunomodulation with TGF-β1 PLGA MPs Resulted in Localized Immunotolerance. **(A)** Frequency of Foxp3^+^ CD4^+^ T cells (peripheral Tregs) in spleens and lymph nodes of non-transplanted naive controls (black dots, n=3) and the long-term allogeneic islet graft survivors (blue triangles, n=3) treated with TGF-β1 PLGA MPs. **(B)** Representative flow cytometric gating used to quantify CD8^+^ T cells response of the long-term islet graft survivors to different donor antigens. **(C)** Using MLR assay, splenocytes isolated from non-rejecting allogeneic islet graft survivors (blue bars, n=3) with TGF-β1 PLGA MPs treatment demonstrated normal immune responses to syngeneic donor (C57BL/6), allogeneic donor (Balb/C) and the third-party MHC-mismatched donor (C3H) antigens in a 5-day co-culture, which is statistically equivalent to the responses measured for naïve C57BL/6 responder mice (white bars, n=3). t-test was performed for group comparison, with *p < 0.05 and n.s., not significant.

As summarized in **Figure 8A**, the proportion of Foxp3^+^ CD4 T cells ranged from 7-20%, whereas the percentages of Th1, Th2, and Th17 CD4 T cells were less than 5% in all tested tissues (**Figure S9**). Compared to age-matched (12-15 weeks) naïve non-transplanted mice, long term functioning grafts treated with TGF-β1/PLGA MPs showed no difference in Foxp3^+^ CD4^+^ T cells levels in the spleen (*p = 0.76*, t-test) or inguinal (*p = 0.059*, t-test) and brachial (*p = 0.46*, t-test) LNs (**Figure 8A**). Of interest, the number of Foxp3^+^ CD4^+^ T cells in the EFP proximal mesenteric LNs was significantly increased in long-term functional recipients treated with TGF-β1/PLGA MPs (*p = 0.02*, t-test) when compared to age-matched naïve controls. For other T cell lineages, mice with long-term graft survival and particle treatment showed no difference in the frequency of Tbet^+^, GATA3^+^, or RoRγt ^+^ CD4^+^ T cells for all tested tissues, when compared to non-transplanted mice (**Figure S9**), except for increased Tbet^+^ Th1 levels in the brachial LNs (*p = 0.0025*). However, as brachial LNs are distal to the transplantation site, this increase of pro-inflammatory Th1 cells was not suspected to be directly related to the immunomodulation at implant site.

Finally, the potential systemic tolerance to the allogeneic antigen was tested *via* mixed lymphocyte reaction (MLR) with the long-term allograft recipients. Specifically, splenocytes from long-term graft recipients with TGF-β1 PLGA MPs were co-cultured *in vitro* against syngeneic (C57BL/6), allogeneic (Balb/C) or the third-party donor cells (C3H) for five days (64, 65). CD8^+^ T cell responses of the long-term graft recipients to different donors was assessed as the outcome, as measured by the percentage of proliferating granzyme B^+^ viable CD8^+^ T cells (**Figure 8B**).

As shown in **Figure 8C**, CD8^+^ T cells from long-term graft recipients expressed immune responses to BALB/c and C3H third-party stimulation at the levels comparable to the immune responses of non-transplanted naive mice (p > 0.999 for both species). Unstimulated and stimulated (anti-CD3/28 activator beads) control groups validated the MLR platform used in this study. Collectively, these data proved the splenocytes harvested from long-term allograft recipients with TGF-β1/PLGA MPs showed no systemic immunotolerance to the same allo-antigens carried by the accepted islet grafts, revealing the localized nature of any immunomodulation imposed by the TGF-β1/PLGA MPs.

## Discussion

In the past decade, Treg-based therapies have shown increasing potential in dampening both allogeneic and autogenetic immune responses in murine islet transplant recipients and other murine models of graft versus host disease (66–68). The translation from experimental models to clinical transplantation, however, has been disappointing, with challenges in (i) acquiring large quantities of Treg at therapeutic dosage, (ii) the instability of the Treg phenotype and suppressive function post *ex vivo* expansion, (iii) scale up and clinical production, and (iv) the controversy of using polyclonal or antigen-specific Tregs for optimal transplant tolerance (67, 68). Thus, the development of an acellular, off-the-shelf biomaterial-based tolerogenic drug delivery system that could be easily co-transplanted within islet transplants, promote *in vivo* Treg induction, and maintain transplant tolerance is desirable.

In this study, we successfully developed a PLGA microparticle system that provides local delivery of TGF-β1 at the transplantation site. TGF-β1 was selected due to its well characterized function in promoting Foxp3^+^ regulatory T cell differentiation, inhibiting DC maturation, and suppressing CD8^+^ T cell activation when present at appropriate doses (18). Particles were fabricated at the microscale to avoid phagocytosis or convective clearance by host phagocytes, therefore increasing retention of the TGF-β1/PLGA MPs at the graft site (20). Based on particle characterization, TGF-β1/PLGA MPs were monodisperse with a protein entrapment efficiency in-line with previously published reports of similar particles. Specifically, our reported TGF-β1 loading per mg PLGA (0.02 µg/mg PLGA) and entrapment efficiency was within the range of other published methods (0.25 to 40 ng per mg PLGA and 30 - 80% entrapment) (16, 46–49). Also, the final TGF-β1 loading per PLGA for these particles (160 ng per mg PLGA) was in the higher range of reported dosages (4 – 180 ng per mg PLGA) (16, 47, 49). This robust loading density and entrapment efficiency of TGF-β1 into PLGA indicates a potent drug eluting system. The TGF-β1 release profile exhibited an early high release phase, governed primarily by diffusion (69). A second phase of release, controlled by polymer degradation, would have extended TGF-β1 release profile; however, a PLGA degradation-dependent release was not observed for the baseline formulation, even after 30 days.

Attempts to increase the duration of TGF-β1 release by the modification of polymer lactide:glycolide ratio or molecular weight or the addition of osmotic agents had little to no effect, despite published reports on the potential impact of these modifications (15, 51, 52). Protein release from PLGA microparticles is highly influenced by protein charge and size, thus the properties of TGF-β1 may play a role in the lack of efficacy of these approaches. For example, the relatively low isoelectric point of TGF-β1 compared to proteins such as CCL22 may lead to weaker ionic interactions with negatively charged PLGA and thus decrease the likelihood of slowing the impending release (51). Future studies should explore the impact of modulating protein charge or incorporating other carrier proteins to create a more durable TGF-β1 release profile.

With a robust TGF-β1 release in the initial phase of treatment, efficient iTreg induction positively correlated to the TGF-β1 PLGA MPs dosages *in vitro*, with a plateau of approximately 43% conversion of polyclonal iTreg and 25% conversion of antigen-specific iTregs during the 3-day culture window. Additional validation found the Foxp3 expression of iTreg cells was TGF-β1 treatment-specific, with limited expansion of the Foxp3^+^helios^+^ nTreg subset. Treg generation with validated suppressive function by our TGF-β1 PLGA MPs indicates this reported microparticle delivery platform compares favorably to soluble TGF-β1, while providing a means for local delivery within sites not amendable to daily local injections. Furthermore, MP modulated release is more favorable to bolus injections, as it is released within the site over a broader time frame, in lieu of a single daily burst. In addition, although long-term TGF-β1 release was not observed for *in vitro* release profiling, the immunomodulatory impacts of TGF-β1 released over the early release may persist for extended time periods by the potential of TGF-β1 to impart infectious tolerance. Specifically, TGF-β1 can induce peripheral Tregs to convert additional naïve CD4^+^ T cells into Treg in a cell-contact dependent manner to maintain durable immune tolerance (70).

Importantly, since rejection to islet transplant in T1D recipients can be facilitated by both allorejection and recurrent autoimmunity (4, 7), the capacity of TGF-β1 PLGA MPs to generate antigen-specific iTregs could convey additional benefits. Herein, TGF-β1 PLGA MPs were highly efficient in converting OVA-specific OTII CD4^+^ effector T cells to a Foxp3^+^ regulatory phenotype, when compared to unsuppressed controls. The conversion rate for antigen-specific iTregs was generally lower compared to polyclonal iTreg generation, but this was expected based on previous reports (71). The successful induction of antigen-specific iTregs with our particles illustrates the potential of this approach to create a more nuanced immunomodulatory microenvironment, whereby regulatory cells can be generated in a manner specific to the offending antigen. The use of such an approach for islet transplantation into T1D recipients, which exhibit autoimmune memory, could be highly beneficial.

When exploring the use of graft-localized immunotherapies for *in vivo* islet transplantation, it is important to ensure that the tolerogenic drug delivery system does not impose adverse effects on pancreatic islet viability and function. For islet transplantation, numerous effective immunosuppressive agents, such as cyclosporine and tacrolimus, are known to negatively impact islet and beta-cell function and survival (72, 73). Furthermore, some agents may be safe for systemic delivery, but impart detrimental effects when delivered locally (39). Screening of the TGF-β1/PLGA MPs with pancreatic islets confirmed no adverse impacts on pancreatic islet metabolic activity, viability, and functional glucose responsiveness. This provided an avenue for the local co-transplantation of islets with these microparticles.

The co-transplantation of allogeneic islets with TGF-β1/PLGA MPs into chemically-induced diabetic recipients resulted in no significant delay in the timeline to normoglycemia. This confirms the compatibility of local TGF-β1 release and PLGA microparticles, including their degradation by-products, on islet engraftment, at the dosage tested. Graft analysis also did not indicate elevated fibrotic deposition, a common deleterious effect of elevated TGF-β1 levels (28). Thus, this work established a future for the integration of localized drug-delivery microparticle depots within the islet transplant microenvironment.

The local delivery of TGF-β1 can be achieved through variable formats, from macro, micro, to nano-scale, with all approaches exhibiting advantages and disadvantages. While TGF-β1-releasing macroscale scaffolds have demonstrated benefits in local modulation and provide the advantage of 3-D structure (17), their structured format restrict adaptation to a specific implant size. On the nanoscale, PLGA nanoparticles loaded with interleukin-2 and TGF-β1 observed elevated iTreg (16); however, the scale of these particles incur issues associated with off-target delivery, enhanced phagocytosis, and the need for surface modification to enhance targeting and retention. The microscale format, while challenged in the delivery of high doses and duration of release, exhibit the advantages of injectability, limited phagocytosis, and site retention. In addition, microparticles containing different agents may be easily integrated by creating particle cocktails, e.g. TGF-β1, IL-2, and immunosuppressant rapamycin (15), and/or combining with antigen-presenting cell targeted particle for enhanced Treg conversion, recruitment, and/or stability for improving islet transplantation conversion in islet transplantation models (46). Due to the complex immunological responses initiated following human allogeneic islet transplantation, it is likely that a multi-drug approach is needed to induce durable graft acceptance.

Despite establishing the capacity of TGF-β1/PLGA MPs to efficiently induce a regulatory phenotype *in vitro*, the local delivery of TGF-β1/PLGA MPs within the islet transplant site resulted in no significant impact in delaying allograft rejection compared to the islet-only controls. The long-term graft survival (>90 days) of 25% of the allo-islet recipients with these TGF-β1/PLGA MPs was promising, when compared to the reported mean survival time (MST) of islet allografts for this model of 14 days (74); however, this modest shift was not significant. Histological assessment of grafts containing TGF-β1 PLGA MP indicated a local regulatory effect, with a significant increase in local Foxp3^+^CD3^+^ cells, when compared to engrafted allogeneic islets without TGF-β1 PLGA MP treatment. The accumulation of host Foxp3^+^ CD3^+^ cells at the graft site correlates with enhanced allograft survival *via* tolerogenic pathways (75, 76). Also, lymphocytic cell accumulation in long-term functioning grafts has also been observed for systemic immunosuppressive approaches (77, 78), thereby indicating that the localized treatment of TGF-β1/PLGA MPs facilitates a balance between tolerogenic and alloreactive cells. Although the local Foxp3^+^CD3^+^ Treg elevation had been observed, it did not convert to significant graft protection, indicating the amount and efficacy of the *in vivo* induced Treg cells, either related to the low dosage of PLGA particle releasing TGF-β1 applied in this study or the polyclonal specificity of the *in vivo* iTregs, may be insufficient to generate therapeutic benefits and graft protection. Comparing the delivered MP TGF-β1 dose to that supplied using a macroscale scaffold implant, which delivered an estimated 1000-fold higher TGF-β1 amount into the local microenvironment, further supports this hypothesis (17). For future investigation, synergistic therapy to boost up allogeneic antigen presentation, the *in vivo* kinetic study of Treg cells and cytokine secretion at and/or peri-transplant site, for both naïve or TGF-β1 PLGA MPs treated allo-islet recipients, will be beneficial for improved *in situ* immunomodulation.

To gain better insight into the impacts of TGF-β1/PLGA MPs on the immune system of recipients, CD4^+^ T cell phenotyping and mixed lymphocyte reactions were performed to capture potential systemic tolerance in the long-term allografts survivors. CD8^+^ T cells from TGF-β1/PLGA MPs-treated long-term graft recipients (C57BL/6J; H-2^b^) generated immunoreactivity to allogeneic (Balb/c; H-2^d^) and the third-party (C3H; H-2^k^) stimulation comparable to the response measured from naïve animals, demonstrating the lack of systemic tolerance related to the particle treatment. Phenotyping of CD4^+^ T cells in spleen and lymph nodes of TGF-β1/PLGA MPs-treated recipients, however, found an increased regulatory T cell presence only within lymph nodes draining from the transplant site. These results indicate that TGF-β1 PLGA MPs generate elevated local iTregs without systemic effects. Globally, Tbet^+^ (Th1) and GATA3^+^ (Th2) CD4^+^ T cells, which are known to be important facilitators of both acute and chronic allograft rejection (79–81), and the pro-inflammatory Th17 phenotype (RoRγt^+^ CD4^+^) cells were not elevated in the LNs or spleens of long-term engrafted mice with TGF-β1 PLGA MPs treatment, indicating limited detrimental impacts of this local therapy.

Beyond CD4^+^ T cell modulation, local TGF-β1 could also impart broader effects. For example, the local release of TGF-β1 from macro-scaffolds resulted in decreased leukocyte infiltration, macrophage maturation, and pro-inflammatory cytokine levels at the local graft site at early time points (3 to 7 days post-transplantation). Thus, future work may seek to expand immunophenotyping to multiple cell types and time points.

Overall, this study established a successful PLGA microparticle platform for ease in co-localization within extrahepatic transplant sites for islet implantation. TGF-β1 release from PLGA MPs was effective in generating suppressive T regulatory cells *in vitro* and providing a means to locally deliver this agent into the islet graft site without detrimental effects. Local release of this monotherapy at this dosage, however, was insufficient in substantially delaying graft rejection, when compared to untreated controls. As such, future work should investigate the potential of this PLGA MP approach to locally deliver multiple immunosuppressive agents, such as CCL22 (63), or to combine local TGF-β1 release with modest systemic immunotherapy.

## Conclusion

In conclusion, this study developed a TGF-β1 releasing PLGA microparticle platform that supported localized drug delivery, robust polyclonal and antigen-specific iTreg generation *in vitro*, and a potential for modulating local immune responses to allogeneic islet implants within extrahepatic transplantation sites. Though no significant improvement in graft efficacy was achieved, the co-transplantation of TGF-β1 PLGA MPs along with allogeneic islet grafts resulted functional engraftment and an elevated presence of induced T regulatory cells *in vivo*, implicating a local alteration of immune cell phenotype. Together, this work established the feasibility of a local immunomodulatory biomaterial delivery system that is compatible with islet engraftment.

## Data Availability Statement

The raw data supporting the conclusions of this article will be made available by the authors, without undue reservation.

## Ethics Statement

The animal study was reviewed and approved by IACUC at the University of Florida, Gainesville, FL.

## Author Contributions

YL, AF, AB, BK, and CLS developed the concept. YL, AF, and CLS. designed the study and analyzed data. YL, AF, YR, CS, MS, and SB conducted experiments and acquired data. J-PL and YR conducted scanning electron microscopy. YL, AF, IL, and MS conducted islet isolations and allogenic islet transplantations. All authors contributed to the article and approved the submitted version.

## Funding

This work was supported by NIH grant DK100654 and DK126413.

## Conflict of Interest

The authors declare that the research was conducted in the absence of any commercial or financial relationships that could be construed as a potential conflict of interest.

## Abbreviations

APC, Antigen Presenting Cell; BSA, Bovine serum albumin; ELISA, Enzyme linked immunosorbent assay; GSIR, Glucose stimulated insulin response; iTreg, Induced regulatory T cells; LAL, Limulus amoebocyte lysate; MHC, major histocompatibility complex; MLR, Mixed lymphocyte reaction; MPs, microparticles; nTreg, Natural regulatory T cells; OVA, Ovalbumin; PBS, Phosphate buffered saline; PI, Proliferation index; PLGA, Poly(D,L-lactide-co-glycolide); pTreg, Peripheral regulatory T cells; PVA, polyvinyl alcohol; STZ, streptozotocin; T1D, Type 1 Diabetes.
