# Supplementary material for: Immunosuppressive PLGA TGF-β1 Microparticles Induce Polyclonal and Antigen-Specific Regulatory T Cells for Local Immunomodulation of Allogeneic Islet Transplants
Source: Front Immunol. 2021 May 27;12:653088. doi: 10.3389/fimmu.2021.653088 (PMC8190479; doi:10.3389/fimmu.2021.653088)
Supplement: Supplementary file 1 [file DataSheet_1.docx]

**Immunosuppressive PLGA TGF-β1 Microparticles Induce Polyclonal and Antigen-Specific Regulatory T Cells for Local Immunomodulation of Allogeneic Islet Transplants**

Ying Li^#1-2^, Anthony W. Frei^#1^, Irayme M. Labrada^1^, Yanan Rong^1^, Jia-Pu Liang^1^, Magdalena M. Samojlik^1^, Chuqiao Sun^1^, Steven Barash^1^, Benjamin G. Keselowsky^1-3^, Allison L. Bayer^4-5^, Cherie L. Stabler*^1-3^.

^1^J. Crayton Pruitt Family Department of Biomedical Engineering, University of Florida, Gainesville, FL, USA.

^2^Graduate Program in Biomedical Sciences, College of Medicine, University of Florida, Gainesville, FL, USA.

^3^University of Florida Diabetes Institute, Gainesville, FL, USA.

^4^Diabetes Research Institute, University of Miami, Miami, FL, USA.

^5^Department of Microbiology and Immunology, University of Miami, Miami, FL, USA.

**SUPPLEMENTAL DOCUMENTS**

**Supplemental Methods**

***Endotoxin Quantification***

Endotoxin levels of TGF-β1/PLGA MPs were tested on the supernatant of the particles. Briefly, 1 mg particles were incubated in 200 µL sterile cell culture water (Corning) in a 96-well plate for 48 hours, after which the water supernatant was collected via centrifugation at 500×g for 5min. The supernatant was tested for endotoxin via LAL Chromogenic Endotoxin Quantitation Kit following the manufacturer's instructions (ThermoFisher).

**Supplemental Tables**

**Table S1**. Variations in PLGA Microparticle Formulation

| **Formulation ID** | **Polymer Viscosity** | **Polymer**  **(L:G Ratio)** | **TGF-beta1**  **(ng/mg PLGA)** | **Additives** |
| --- | --- | --- | --- | --- |
| A | 0.45 dL/g | 50:50 | 20 | - |
| B | 0.2 dL/g | 100:0 | 20 | - |
| C | 0.2 dL/g | 50:50 | 20 | - |
| D | 0.2 dL/g | 75:25 | 20 | - |
| E | 0.45 dL/g | 50:50 | 20 | 2% NaCl |

**Table S2**. List of Antibodies


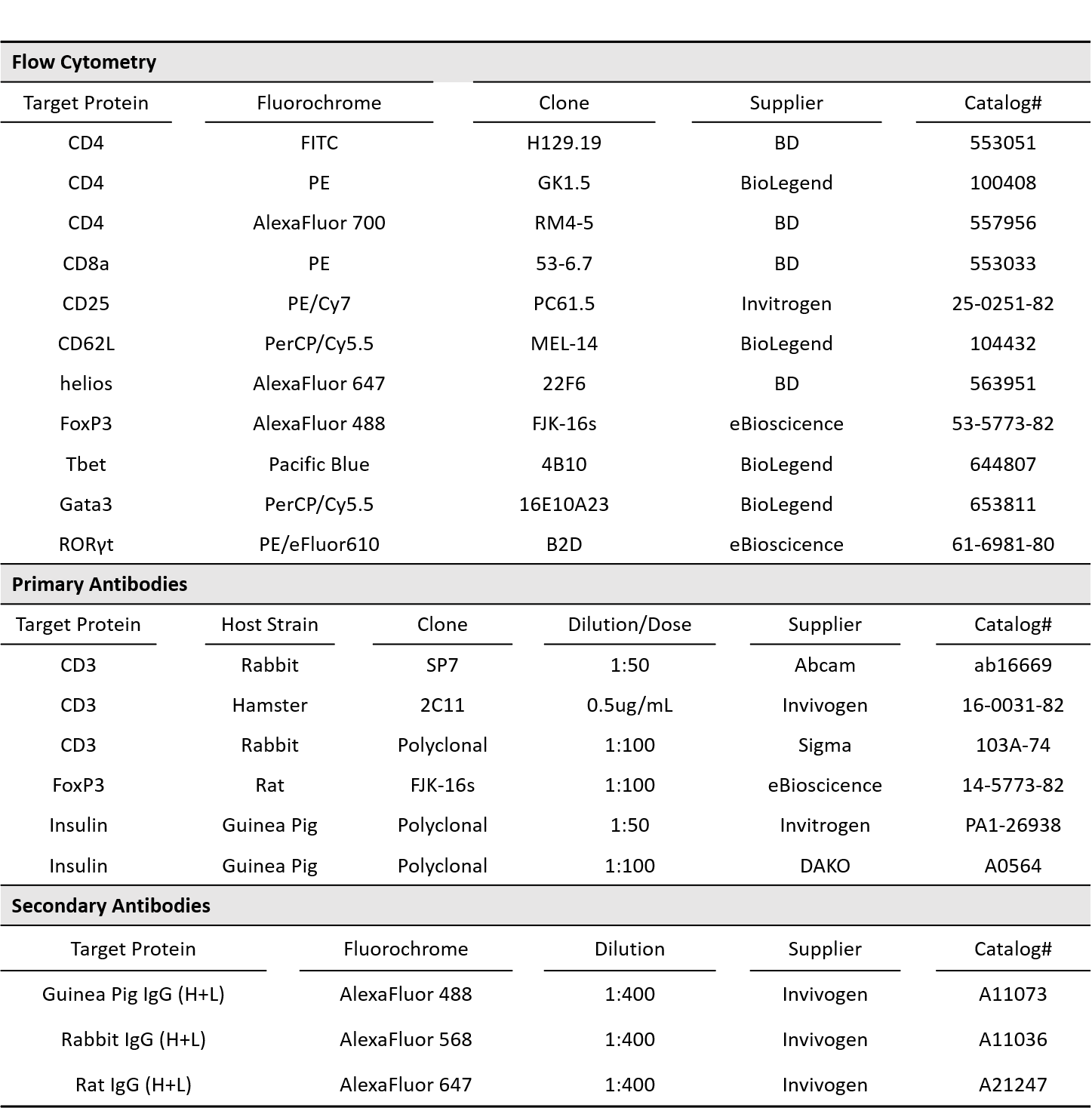


**Table S3.** Endotoxin Quantification of TGF-β1/PLGA Microparticles


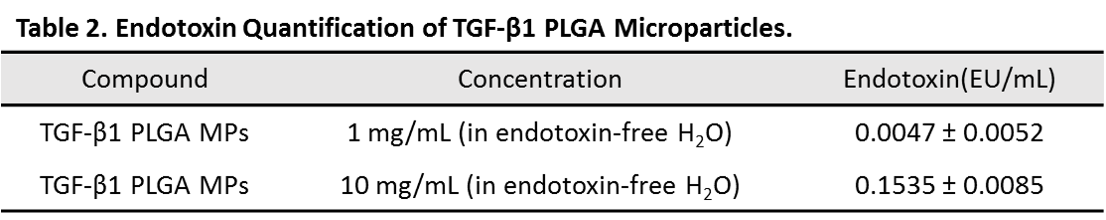


**Table S4.** TGF-β1 Quantification in Treg Conversion Assay.


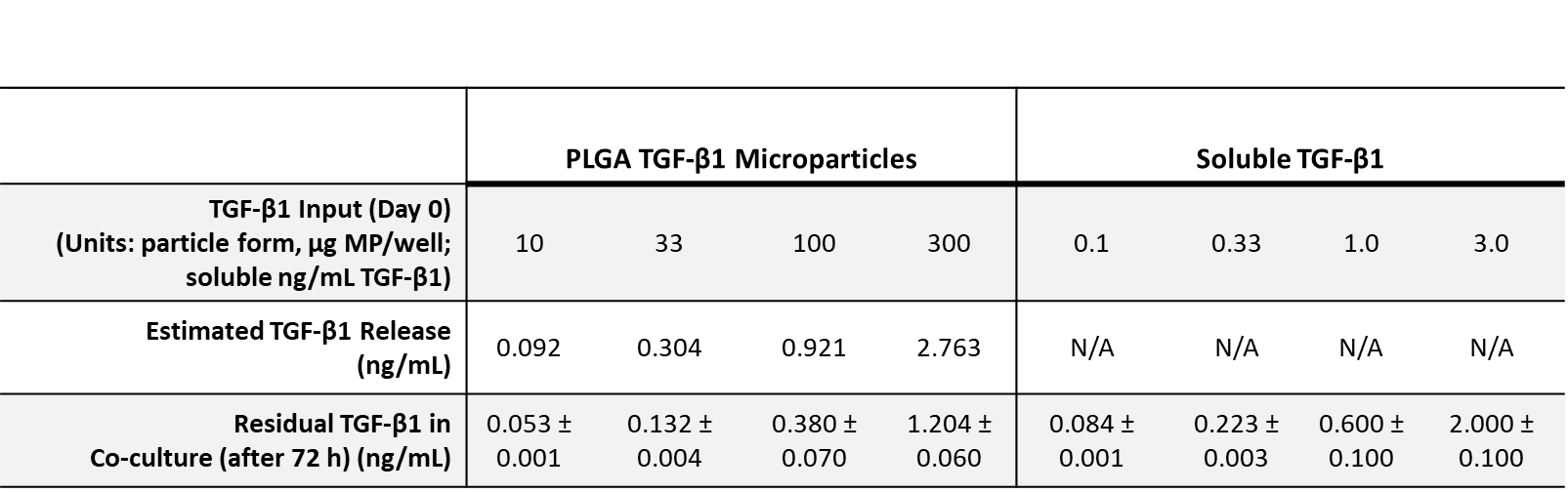


Note: ”Estimated” TGF-β1 released values are theoretical levels based off of kinetic release curves. “Residual” TGF-β1 values are experimentally measured levels within TGF-β1-MP or sTGF-β1 control wells that contained only T cell media

**Supplemental Figures**


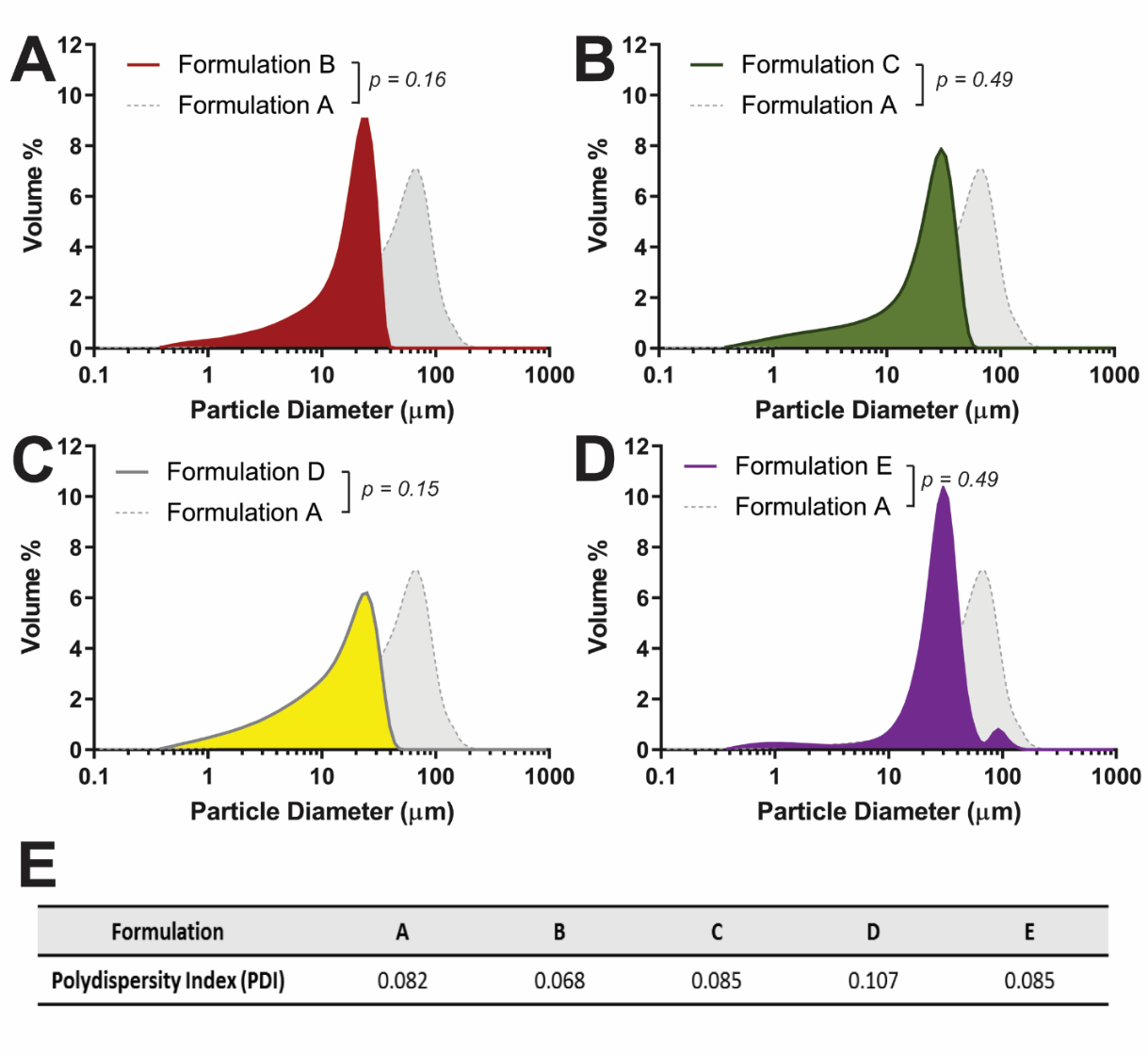


**Figure S1.** **Size Distribution of modified formulations of TGF-β1/PLGA microparticles.** Particle diameter was determined by laser diffraction. Differential volume % from 0 to 2000 µm shown for A) Formulation B, B) Formulation C, C) Formulation D, and E) Formulation E microparticles. E) Polydispersity index for particles of different formulations. Compilation of n = 3 runs per formulation shown.

**
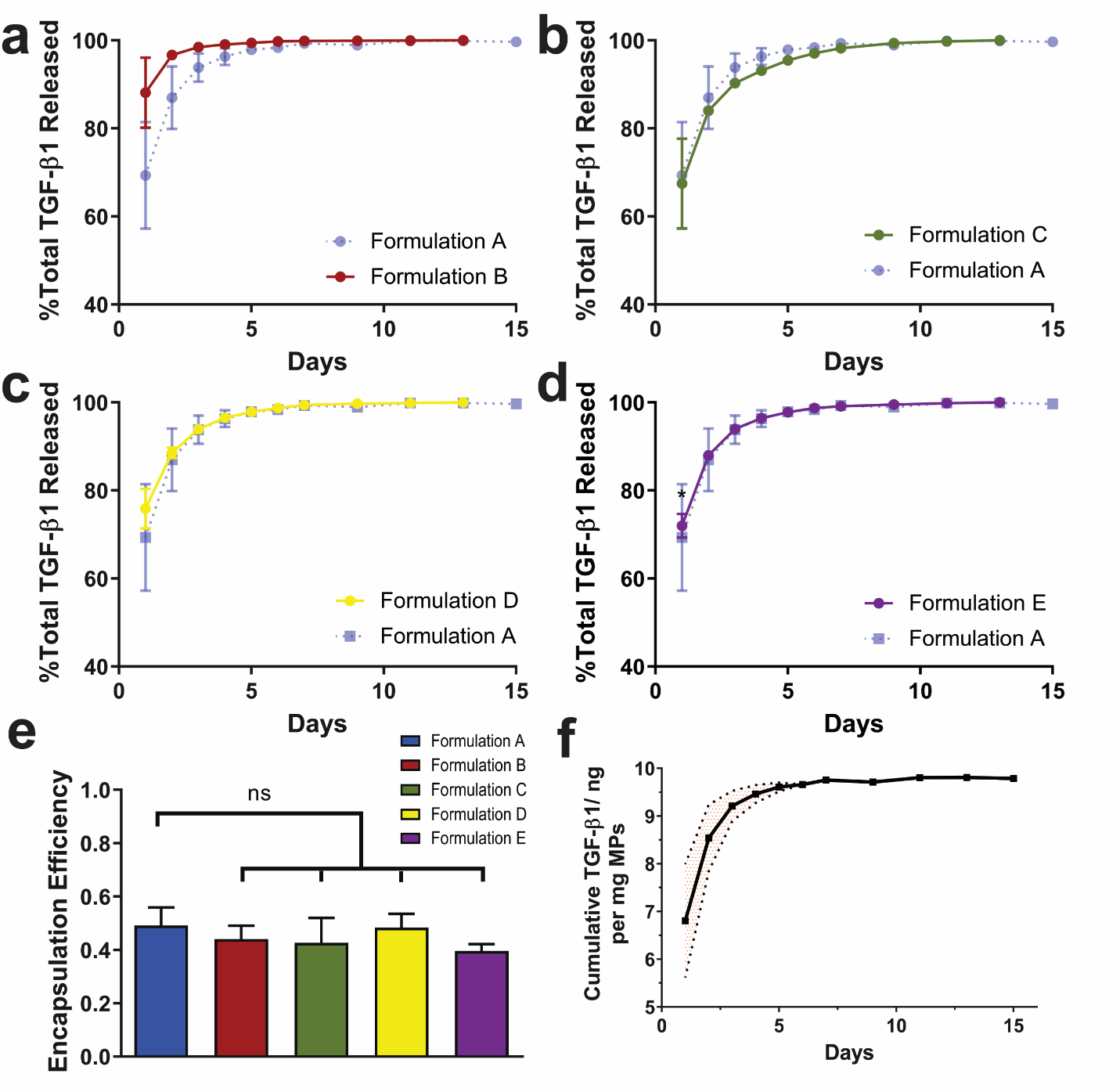
**

**Figure S2. Kinetic Release Profiles and Drug Encapsulation Efficiency of Modified TGF-β1 Microparticle Formulations, compared to baseline Formulation A.** TGF-β1 release profiles of formulation B (a), formulation C (b), formulation D (c), and formulation E (d). All profiles are normalized to total TGF-β1 release over 60 d, with release compared to baseline formulation A (blue dashed line). n=3 per time point. ns = not significant. (e) Summary of TGF-β1 encapsulation efficiency for all five PLGA microparticle formulations. (f) Cumulative release of TGF-β1 ng normalized to particle weight. Mean TGF-β1 release curve (black line) was acquired by averaging four independent (N=4, n=13) studies with particles of different batches, with standard deviation (shading) as shown.


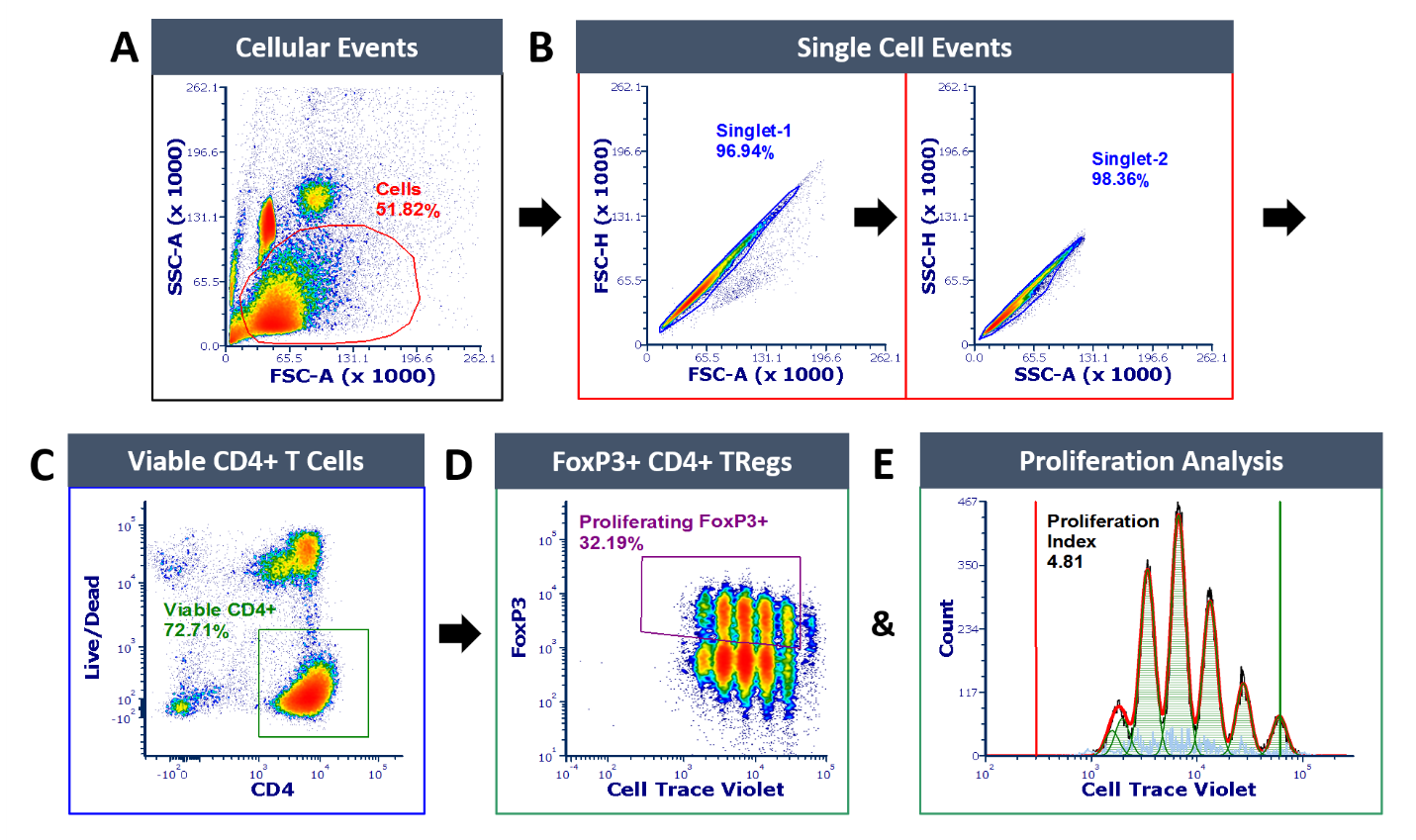


**Figure S3. Representative Flow Cytometry Gating for Enumeration of iTregs.** Sequential gating strategies were applied to quantify the frequency of Tregs induced by PLGA MPs released or soluble TGF-β1. (A) Set a polygon gate to identify the lymphocyte population and exclude debris. (B) Exclusion of cell doublets and aggregation. (C) Viable CD4^+^ T cells were identified as CD4 positive and Live/Dead negative. (D) Proliferating (excluding generation 0) FoxP3 expression on viable CD4^+^ T cells; (E) Proliferation analysis on viable CD4^+^ T cells, where black line = histogram contour of raw data; orange line = fitted data; light blue = noise events; green shaded area = area under the fitted curve; green marker = undivided marker and red marker = background marker. The analysis was performed using FCS Express 6.05 software. The boundary of the gates is determined by isotype controls, fluorescence-minus-one (FMO) controls and the unstimulated control.


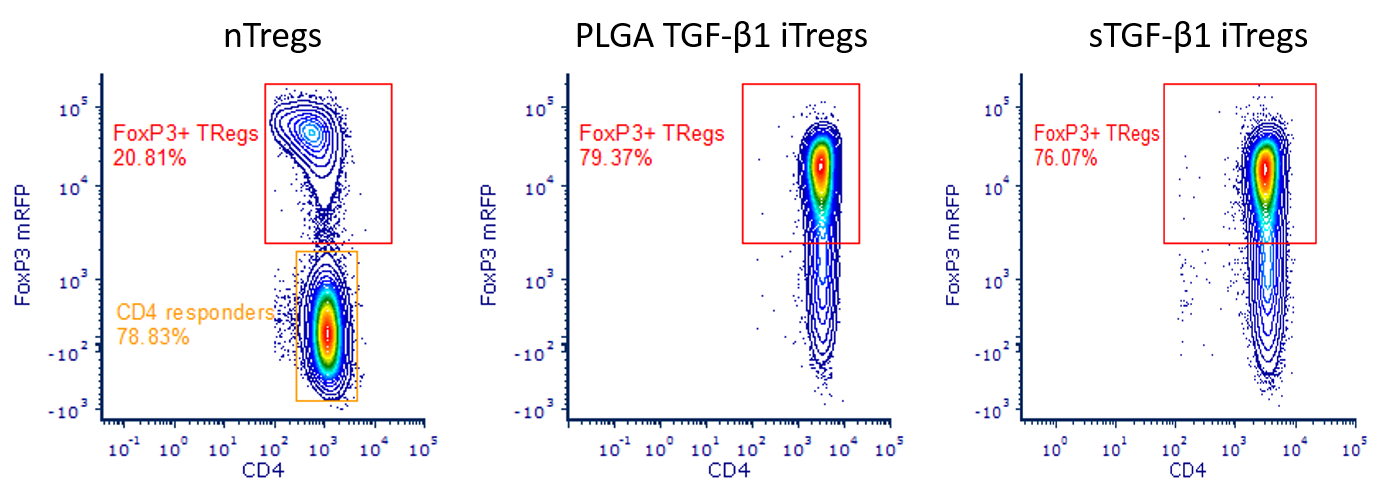


**Figure S4. Representative Flow Cytometric Sorting to Purify FoxP3+ Regulatory T Cells and FoxP3- Responder T cells.** Freshly isolated B6-FIR lymphocytes and B6-FIR naïve CD4^+^ T cells post Treg conversion were used for sorting. Cell debris and aggregates were excluded by serial gating. Then viable CD4^+^ T cells were identified base on signals of CD4-FITC and Live/Dead IR dye staining. FoxP3 expression was identified by the endogenous mRFP signal. CD4^+^FoxP3^+^ cells of the different sources (as noted) were sorted out as suppressors, while the CD4^+^FoxP3^-^ cells were used for responder cells. Gating boundaries were determined based on fluorescence-minus-one (FMO) controls and the unstimulated control with proper compensation. Three independent sorts were performed using BD FACSAria II Sorter with an efficiency of over 98%.


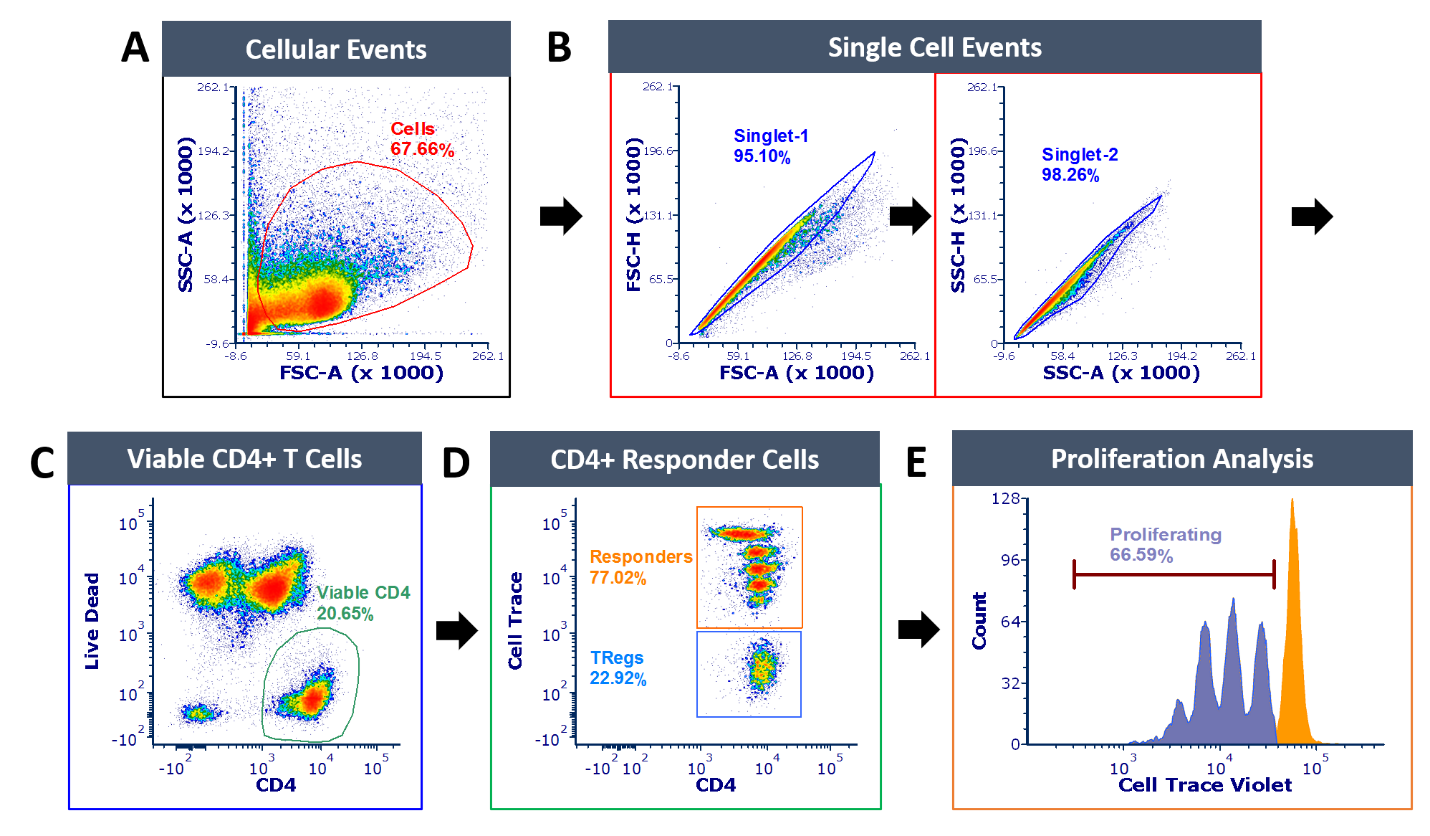


**Figure S5. Representative Flow Cytometry Gating for Treg Suppression Assay.** Sequential gating strategies were applied to assess the suppressive function of Tregs induced by PLGA MPs released or soluble TGF-β1, compared to natural Tregs. (A) Set a polygon gate to identify the lymphocyte population and exclude debris. (B) Exclusion of cell doublets and aggregation. (C) Viable CD4^+^ T cells were identified as CD4 positive and Live/Dead negative. (D) CD4^+^ responder cells were identified as CD4 and CellTrace Violet double positive. (E) Frequency of proliferating (excluding generation 0) CD4^+^ responder cells were quantified using histogram. The analysis was performed using FCS Express 6.05 software. The boundary of the gates is determined by isotype controls, fluorescence-minus-one (FMO) controls.


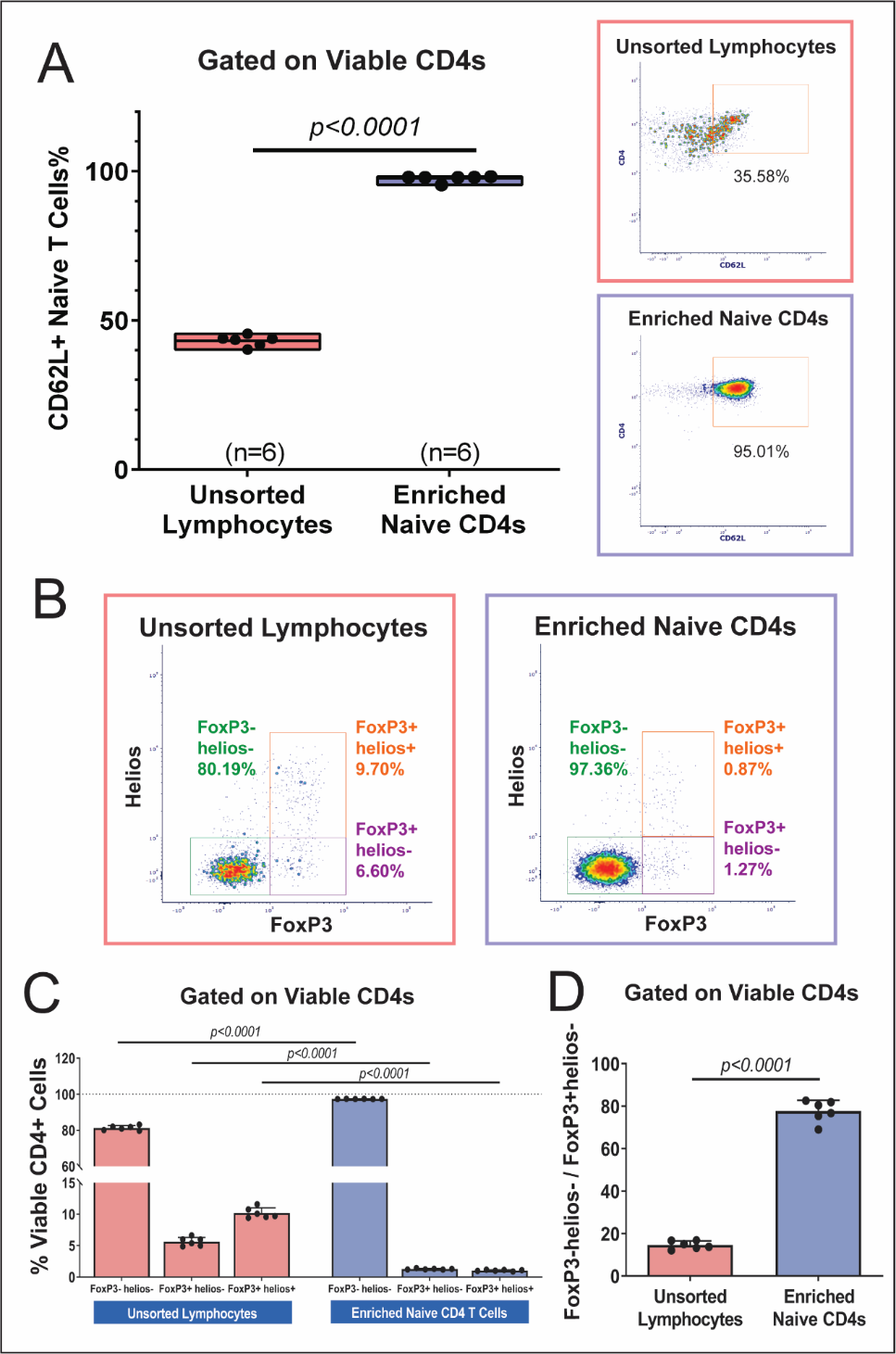


**Figure S6. Purity of Magnetically Sorted Naïve CD4^+^ T Cells for Treg Conversion Assay.** (A) Frequency of naïve CD4^+^ T cells (LiveDead^-^CD4^+^CD62L^+^) before (pink bar) and after (blue bar) magnetic purification with StemCell negative selection kit, with representative cytometric density plots. (B) Representative gating of CD4^+^FoxP3^-^helios^-^, CD4^+^FoxP3^+^helios^+^, and CD4^+^FoxP3^+^helios^-^ cells before (pink box) and after (blue box) magnetic purification with (C) summarized quantification (n=6). (D) Ratio between Foxp3^-^helios^-^ and FoxP3^+^helios^-^ CD4^+^ T cells before and after magnetic purification (n=6).


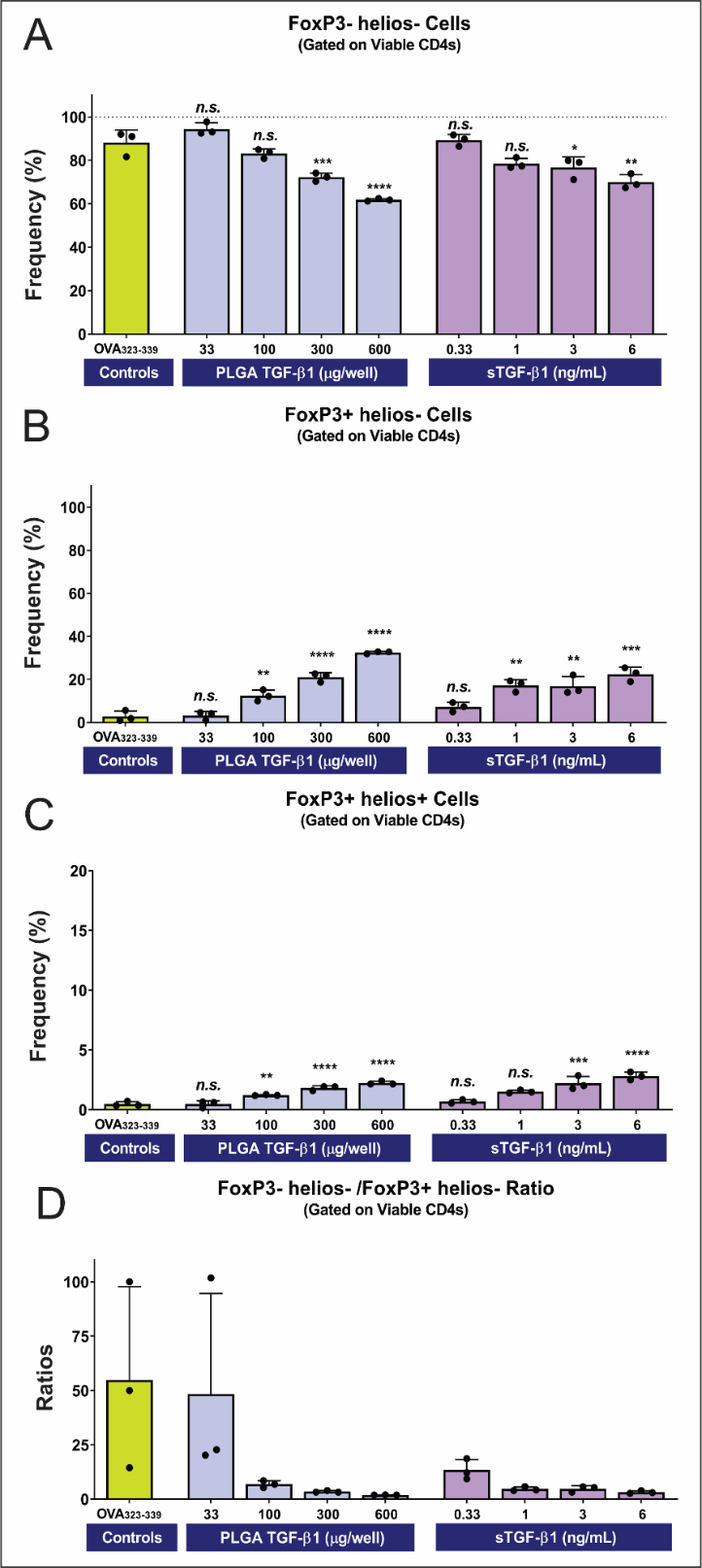


**Figure S7. FoxP3 and helios Expression on CD4+ T cells post in vitro Tregs Conversion using PLGA MPs Releasing or Soluble TGF-β1.** Summary of the frequency of OTII (A) CD4^+^FoxP3^-^helios^-^, (B) CD4^+^FoxP3^+^helios^-^ and (C) CD4^+^FoxP3^+^helios^+^ cells post in vitro conversion by TGF-β1 PLGA MPs (n=3) or soluble TGF-β1 (n=3). Paired Tukey’s test was conducted for mean comparison, with * is used when compared to control group (OVA_323-339_ peptide and APCs only). Statistical significance was determined as ****p < 0.0001, ***p < 0.001, **p < 0.01 and n.s. = not significant. (D) Ratio between FoxP3^-^helios^-^ and Foxp3^+^helios^-^ CD4^+^ T cells before and after the three-day in vitro Treg conversion.


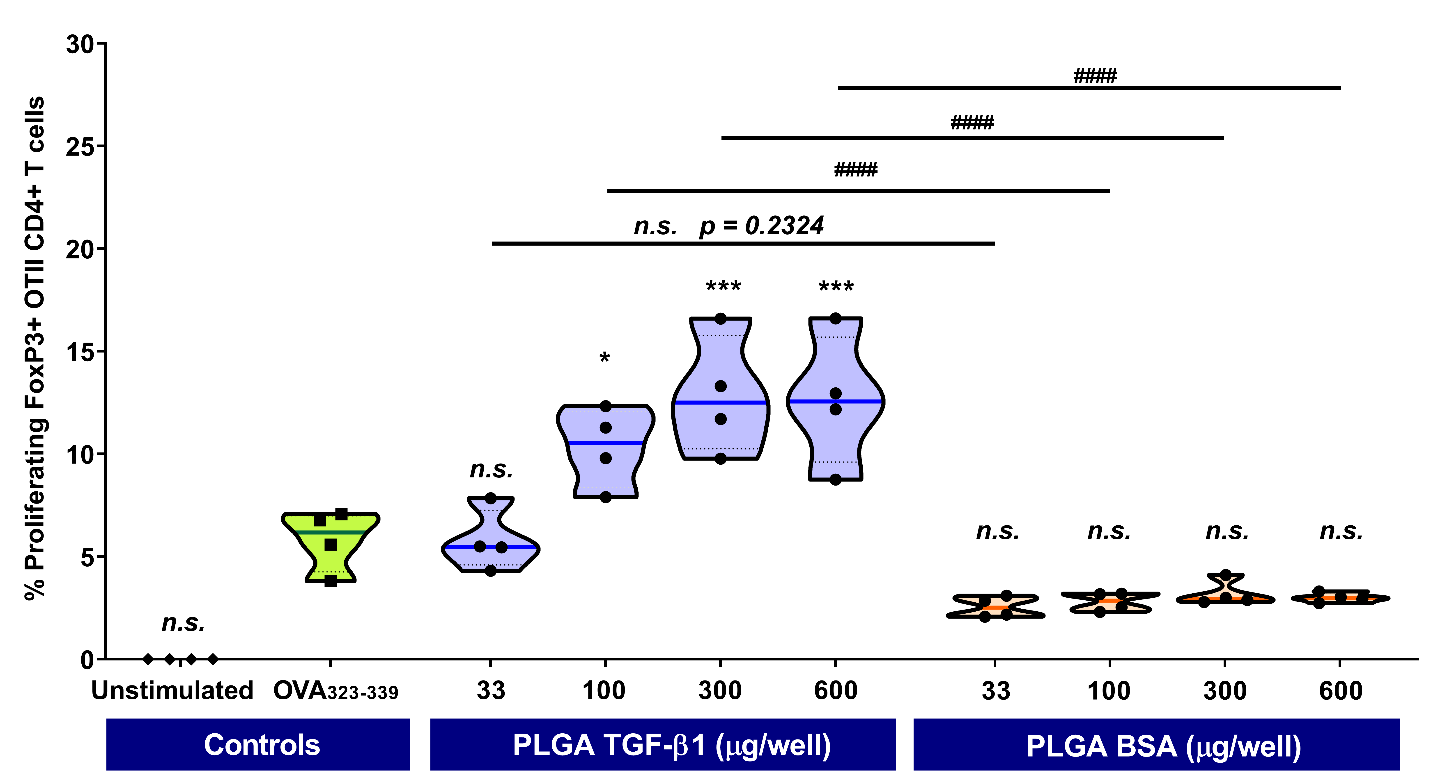


**Figure S8. iTreg Conversion by PLGA MPs is TGF-β1 Specific.** Frequency of OTII CD4^+^FoxP3^+^ iTreg conversion by BSA PLGA MPs vehicle controls compared to TGF-β1 PLGA MPs (n=4).


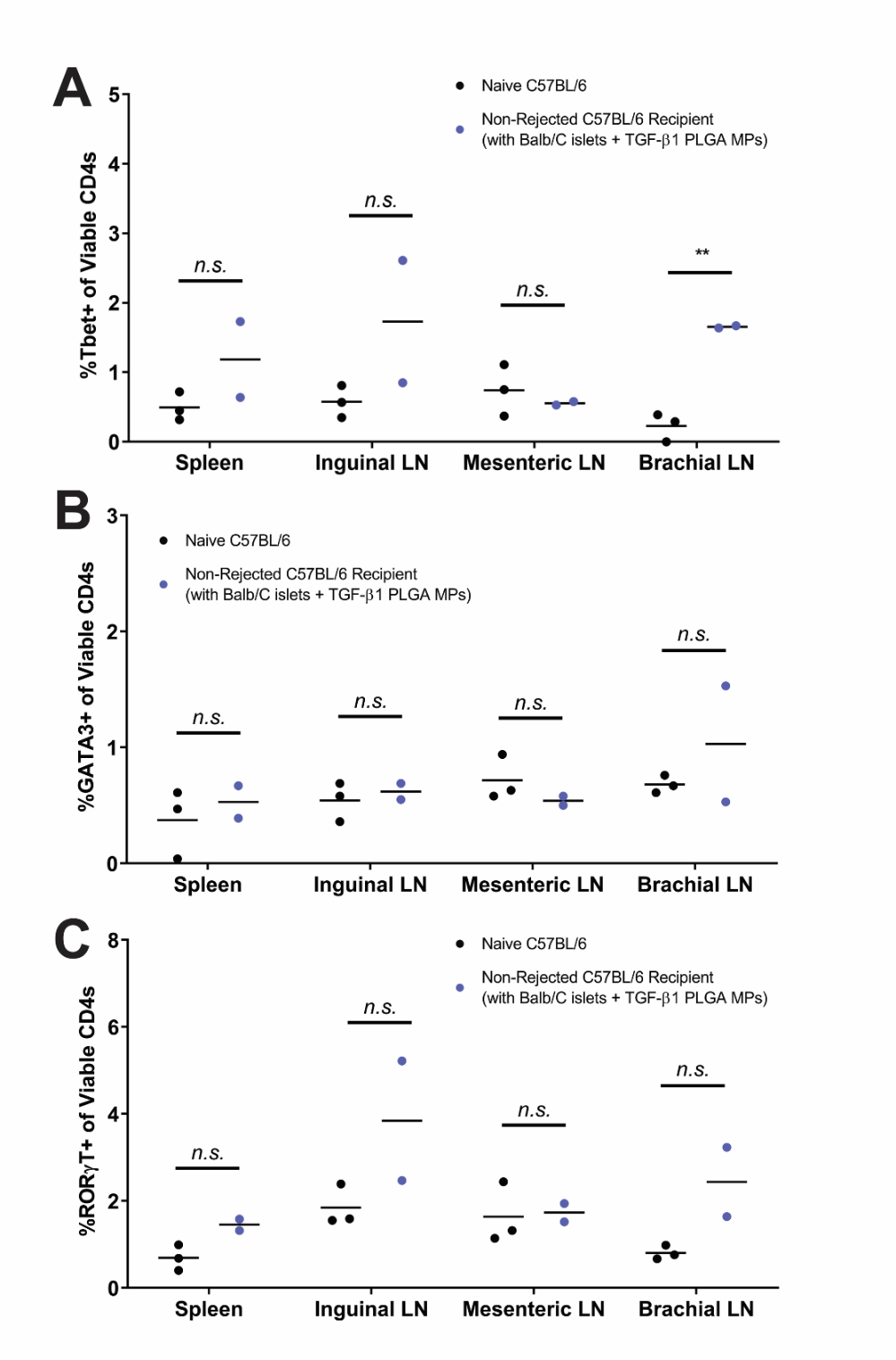


**Figure S9. Phenotype of CD4^+^ T Cells in Spleens and LNs Harvest from Long-term Allogeneic Islet Graft Recipients treated with TGF-β1 PLGA MPs.** Frequency of (A) Tbet^+^, (B) GATA3^+^ and (C) RORγt^+^ CD4^+^ T cells in spleens and lymph nodes of non-transplanted controls (black, n=3) and the long-term allogeneic islet graft survivors (blue, n=2) treated with TGF-β1 PLGA MPs.
